# Tuning and mechanistic insights of metal chalcogenide molecular catalysts for the hydrogen-evolution reaction

**DOI:** 10.1038/s41467-018-08208-4

**Published:** 2019-01-22

**Authors:** James McAllister, Nuno A. G. Bandeira, Jessica C. McGlynn, Alexey Y. Ganin, Yu-Fei Song, Carles Bo, Haralampos N. Miras

**Affiliations:** 1 WestCHEM, School of Chemistry, University of Glasgow, Glasgow, G12 8QQ UK; 2 Institute of Chemical Research of Catalonia (ICIQ), The Barcelona Institute of Science and Technology, Avgda. Països Catalans 16, 43007 Tarragona, Spain; 3 BioISI – BioSystems and Integrative Sciences Institute, Faculdade de Ciências da Universidade de Lisboa, Campo Grande, 1749-016 Lisboa, Portugal; 4 Centro de Química Estrutural, Instituto Superior Técnico, Universidade de Lisboa, Av. Rovisco Pais 1, 1049-001 Lisboa, Portugal; 5 Beijing Advanced Innovation Center for Soft Matter Science and Engineering, State Key Laboratory of Chemical Resource Engineering, Beijing University of Chemical Technology, 100029 Beijing, China; 6 Departament de Química Física i Inorgànica, Universitat Rovira i Virgili, Av. dels Països Catalans, 26, 43007 Tarragona, Spain

## Abstract

The production of hydrogen through water splitting using earth-abundant metal catalysts is a promising pathway for converting solar energy into chemical fuels. However, existing approaches for fine stoichiometric control, structural and catalytic modification of materials by appropriate choice of earth abundant elements are either limited or challenging. Here we explore the tuning of redox active immobilised molecular metal-chalcoxide electrocatalysts by controlling the chalcogen or metal stoichiometry and explore critical aspects of the hydrogen evolution reaction (HER). Linear sweep voltammetry (LSV) shows that stoichiometric and structural control leads to the evolution of hydrogen at low overpotential with no catalyst degradation over 1000 cycles. Density functional calculations reveal the effect of the electronic and structural features and confer plausibility to the existence of a unimolecular mechanism in the HER process based on the tested hypotheses. We anticipate these findings to be a starting point for further exploration of molecular catalytic systems.

## Abstract (web summary)

While hydrogen offers a potential carbon neutral fuel, its production from water using earth-abundant, heterogeneous materials has proven challenging to understand. Here, authors present a series of molecular electrocatalysts based on molybdenum, sulphur, and oxygen for aqueous hydrogen evolution.

## Introduction

Exponential population growth, along with the scale and nature of our energy consumption habits, is a rapidly growing social issue as the global energy demand intensifies our reliance on dwindling fossil fuel reserves with unprecedented environmental consequences. Therefore, growing pressure to pursue alternative energy sources that are both clean and renewable poses a critical challenge to the scientific community^1,2^. In our transition away from the present-day hydrocarbon economy, hydrogen (H_2_) is considered an ideal energy carrier for the future. In particular, sustainable H_2_ production from water splitting has attracted tremendous attention due to its high-energy density and potential utilisation without carbon emissions^3^. Yet the development of commercially viable energy production systems based on electrocatalysis or photocatalysis of aqueous media is currently limited by the availability of low-cost catalysts needed to overcome the minimum thermodynamic potential. For example, platinum-based catalytic systems are the most efficient hydrogen evolution reaction (HER) electrocatalysts^4^. However, low abundance and high cost of precious metals ultimately restricts their large-scale commercial applications. Therefore, the development and pursuit of cheap, noble-metal free electrocatalysts is highly desirable.

Metal chalcogenides have attracted tremendous research interest as they play a fundamental role in a wide range of important energy generation and storage applications including catalysis, photovoltaics, batteries and artificial photosynthesis systems^5^. More specifically, molybdenum disulphide (MoS_2_) is one of the most widely used catalysts in industry as the standard for hydrodesulfurization of petroleum. Additionally, it has been demonstrated that MoS_2_ is a promising and cost-effective alternative to platinum for electrochemical and photochemical generation of hydrogen from water. The main source of catalytic activity of this layered material stems from the triangular MoS_2_ edges of the structure. Theoretical and experimental studies have identified that catalytic activity towards the HER is directly proportional to the number of unsaturated sulphur atoms at the edges of MoS_2_. Moreover, density functional theory (DFT) calculations of nanoparticulate MoS_2_ have shown that only the edge sites of the MoS_2_ have a suitable Gibbs free energy of hydrogen adsorption (Δ*G*_H_ = 0.08 eV), whereas the (0001) basal planes are relatively inert^6–8^. Thus, maximising the number of active edge sites is one of the key parameters for enhancing the catalytic performance. However, preparing a crystalline phase with large catalytically active edge dimensions is extremely challenging. Most importantly, fine tuning 2D nanoparticulate materials in an effort to optimise the physical and chemical interactions between the catalytic phase (HER catalyst) and the substrate (in this case H^+^ cations) is neither facile nor easily designed due to constraints imposed by the structure^9^. On the other hand, appropriate molecular species fit this bill extremely well since they have well-defined structures and it is possible to employ design principles for generating species containing modular and catalytically active sites.

One promising strategy is the design of molybdenum sulphide nanoclusters which mimic the catalytically active sites found in MoS_2_. These molecules have the unique property of exposing a high degree of active edge sites leading to improved catalytic activity. In recent years, efforts focused on developing this research strand have provided information on potential reaction pathways that take place during the process. For example, in 2012, Karunadasa et al. reported a MoS_2_ edge-site mimic complex which is able to act as a homogeneous catalyst for hydrogen generation^10^. A couple of years later in 2014, Kibsgaard et al. followed by Huang et al. in 2015, demonstrated the catalytic activity of the [Mo_3_S_13_]^2–11^ and [Mo_2_S_12_]^2–12^ cluster-based heterogeneous catalysts which exhibited highly promising HER activity. Interestingly, Garrett, Wu et al. reported very recently their studies on a tunable family of mononuclear complexes with the general formulae, [MoO(S_2_)_2_L_2_]^–^ and [MoO(S_2_)_2_L], where they demonstrated quite elegantly the electronic influence of the organic component on the metal centre and subsequently on the catalytic hydrogen evolving process in homogeneous systems^13–15^. In general, examples of molecule and cluster-based catalysts which demonstrate activity for the HER are relatively limited to the research efforts conducted recently and described above^10,11^.

In this work, we explore the implementation of new design principles for the development of efficient molecular HER catalysts. Specifically, we aim to investigate the effects of stoichiometric and structural control resulting from the appropriate choice of the chalcogen elements and their ratio (e.g. O^2−^/S_2_^2−^, O^2−^/S_2_^2−^/S_4_^2−^) within the coordination sphere of earth abundant transition metals (e.g. Mo or W). We evaluate the performance of these molecular catalysts for the HER while we propose potential mechanistic pathways in relation to the interaction between catalysts and substrate. More specifically, we report the preparation and investigation of three oxothiometalate molecular species and demonstrate their highly efficient electrocatalytic activity towards the HER. Notably, these results suggest that the design of MoS_2_ molecular analogues represents an attractive strategy for preparing next-generation HER electrocatalysts, potentially competing with current state-of-the-art Pt-based ones.

## Results

### Synthesis of the molecular catalysts

**Fig. 1:**
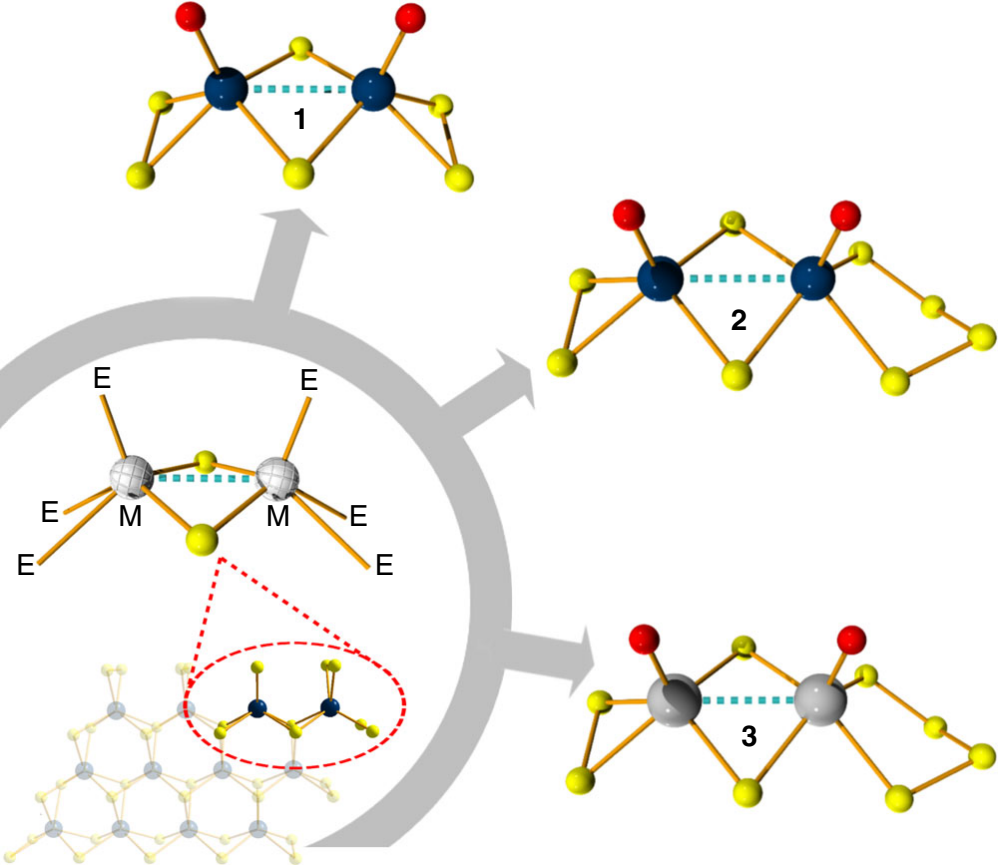
Ball-and-stick representation of the anionic dimeric species [Mo_2_O_2_(*µ*-S)_2_(S_2_)_2_]^2–^ **1**, [Mo_2_O_2_(*µ*-S)_2_(S_2_)(S_4_)]^2–^ **2** and W_2_O_2_(*µ*-S)_2_(S_2_)(S_4_)]^2–^ **3**. Where M: Mo or W and E: chalcogen atom. Colour code: Blue, Mo; Grey, W; yellow, S; red, O; green, N; black, carbon; Counterions are omitted for clarity

The dimeric nanoclusters, [Mo_2_O_2_(*µ*-S)_2_(S_2_)(S_*x*_)]^2–^ (where *x* = 2 or 4), were synthesised by wet-chemical methods in accordance with the original procedures described by Müller et al.^16–18^. The syntheses of both compounds are similar and primarily involve exploiting the thiophilicity of molybdenum by exposing an alkaline molybdate solution to sulfurizing conditions (Supplementary Figure 1). The tungsten analogue, [W_2_O_2_(*µ*-S)_2_(S_2_)(S_4_)]^2–^, was synthesised following a similar approach^19,20^. Each method yielded orange-red crystals which were characterised by single crystal and powder X-ray structural analysis, FT-IR, PXRD and high-resolution electrospray ionisation mass spectroscopy (ESI-MS). Subsequently, the compounds were investigated as potential electrocatalysts to produce H_2_ gas from acidic media where they were found to exhibit excellent activity towards the HER. The oxothiometalate compounds were synthesised open to air at hundreds of gram scale, following a facile two-step process and utilising low-cost materials, thus illustrating their potential for scalability (Fig. 1).

Single crystal X-ray diffraction (XRD) analysis shows that [(CH_3_)_4_N]_2_[Mo_2_O_2_(*µ*-S)_2_(S_2_)_2_] **1** crystallises in the orthorhombic space group *Pba2* with unit cell dimensions of *a* = 15.430(1), *b* = 24.542(1), *c* = 10.8423(8) Å. Figure 1 displays the crystal structure of the [Mo_2_O_2_(*µ*-S)_2_(S_2_)_2_]^2–^ anion. The anion is constructed by two equivalent molybdenum centres that are mutually linked by two sulphur bridges, and a Mo^V^–Mo^V^ separation of 1.684(3) Å (single bond). In addition, the coordination sphere of each molybdenum centre is completed by a terminal disulphide (S_2_) group, with an average S–S bond distance of 2.079(2) Å, a terminal oxo group (Mo = O) 1.684(3) Å and two sulphide bridging atoms with [Mo–(*μ*-S)]_av_ of 2.326 Å. The overall charge of the compound is compensated by two tetramethyl-ammonium, (CH_3_)_4_N^+^, cations (Supplementary Figure 2).

[(CH_3_)_4_N]_2_[Mo_2_O_2_(*µ*-S)_2_(S_2_)(S_4_)] **2** (Fig. 1) crystallises in the monoclinic space group *P21/c* with unit cell dimensions of *a* = 20.661(7), *b* = 9.769(3), *c* = 12.428(4) Å. Structurally the dimeric cluster is similar to [Mo_2_O_2_(*µ*-S)_2_(S_2_)_2_]^2–^, the main difference being that one terminal S_2_ group has been replaced by a tetrasulfide (S_4_) group. In a similar manner, the coordination spheres of Mo1 and Mo2 centres are completed by a terminal disulphide (S_2_) and a tetrasulfide (S_4_) group, respectively, with average S–S bond distances of 2.077(2) and 2.054(2) Å (Supplementary Figure 3). Finally, the Mo=O bond lengths are found to be 1.682(4) and 1.692(4) Å, while the two sulphide bridging atoms are located at 2.301(1) and 2.360(1) Å away from Mo1 and Mo2 centres, respectively. Bond valence sum (BVS)^21^ calculations confirmed the oxidation state of the molybdenum centres which was found to be +V (BVS_av_ = 5.08 **1** and 4.80 **2**). All the bond lengths observed in **1** and **2** are in agreement with previously reported examples^22,23^.

[(CH_3_)_4_N]_2_[W_2_O_2_(*µ*-S)_2_(S_2_)(S_4_)] **3** (Fig. 1), crystallises in the monoclinic space group *P21/c* with unit cell dimensions of *a* = 20.881(2), *b* = 9.778(1), *c* = 12.338(1) Å. Structurally the dimeric cluster is similar to [Mo_2_O_2_(*µ*-S_2_)(S_2_)_4_]^2-^. In a similar manner, the coordination spheres of W1 and W2 centres are completed by a terminal disulphide (S_2_) and a tetrasulfide (S_4_) group, respectively, with average S–S bond distances of 2.083(2) and 2.073(2) Å. Finally, the W=O bond lengths are found to be 1.709(6) and 1.715(7) Å, while the two sulphide bridging atoms are located at 2.297(2) and 2.368(2) Å away from W1 and W2 centres, respectively. Bond valence sum (BVS)^21^ calculations confirmed the oxidation state of the molybdenum centres which was found to be +V (BVS_av_ = 5.01 **3**). All the bond lengths observed in **3** are in agreement with previously reported examples^20,24,25^. Additionally, the compounds were characterised in solid state by FT-IR (Supplementary Figure 4) while the purity of the isolated crystalline phases were confirmed by powder XRD (Supplementary Figure 5). The powder patterns recorded for the [(CH_3_)_4_N]_2_[Mo_2_O_2_(*µ*-S)_2_(S_2_)(S_*x*_)] (*x* = 2 or 4) and [(CH_3_)_4_N]_2_[W_2_O_2_(*µ*-S)_2_(S_2_)(S_4_)] compounds were in good agreement with the simulated powder XRD based on the single crystal XRD data.

### Solution stability and characterisation

**Fig. 2:**
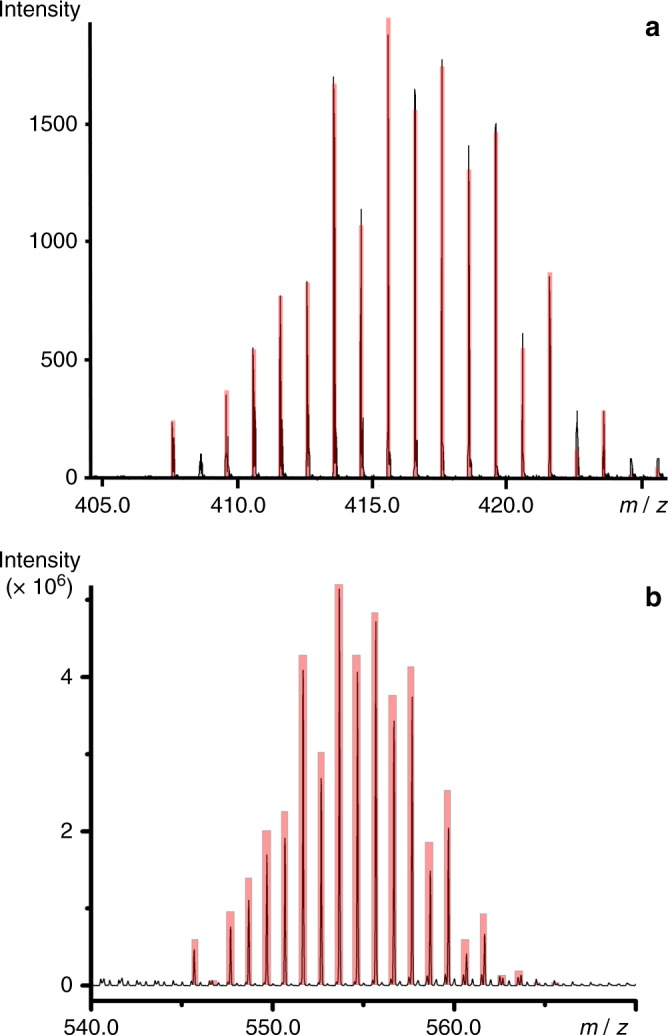
Mass spectrometry. Negative ion mass spectrum in CH_3_CN solution of **a **{Mo^VI^Mo^V^O_2_S_6_}^–^ **1** and** b** {[(CH_3_)_4_N][Mo_2_^VI^O_2_S_8_]}^–^ **2**. The distribution envelopes are centred at *m/z* ca. 415.60 for **1** and 553.67 for **2**. Black line: experimental data, Red bars: simulation of isotope pattern

In an effort to further characterise the isolated compounds, we employed high-resolution electrospray ionisation mass spectrometry (ESI-MS)^26–29^ to determine the composition of the oxothiometalate species in solution. The ESI-MS studies were performed in CH_3_CN. A series of singly charged distribution envelopes observed for **1** and **2**, fall in the regions of 320.6–415.6 and 250.0–580.0*m*/*z*, respectively (Fig. 2). More specifically, the distribution envelopes centred at *m/z* values of 415.60 and 553.67 can be assigned to the intact compounds [Mo^V^Mo^VI^O_2_S_6_]^–^ **1** and {[(CH_3_)_4_N][Mo_2_^VI^O_2_S_8_]}^–^ **2**, respectively.

Molybdenum clusters are generally susceptible to redox processes under the employed ionisation conditions which induces partial fragmentation of the species. Due to the employed ionisation process, an additional series of envelopes have been identified as fragments of the parent species obtained by removal of sulphur atoms due to induced redox processes (Supplementary Figure 6). The change of the oxidation state of the metal centres is due to the ionisation and consecutive ion-transfer process of the charged species and has been observed previously on numerous occasions^30–32^. In a comparable manner, the ESI-MS investigation of **3** was performed in CH_3_CN and revealed a series of singly charged distribution envelopes in the region of 500.0 and 800.0*m*/*z* (Supplementary Figures 7–9). More specifically, the distribution envelope centred at *m/z* value of 729.7 can be assigned to the intact compound {[(CH_3_)_4_N][W_2_^VI^O_2_S_8_]}^–^.

### Electrochemistry of the Mo-based and W-based molecular chalcoxides

**Fig. 3:**
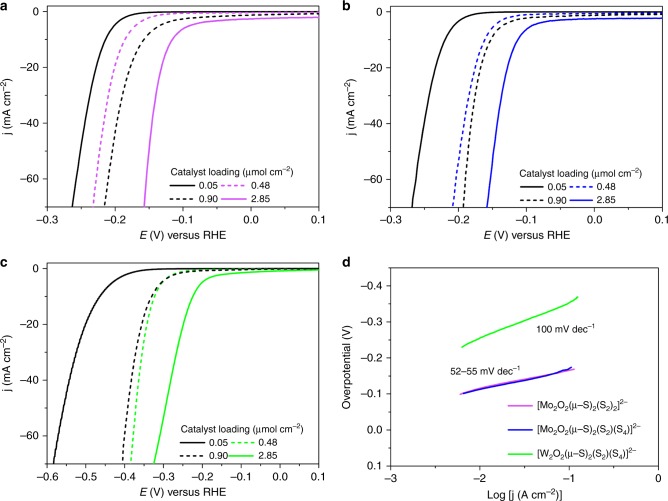
Electrochemical measurements of catalysts **1**–**3** for HER electrocatalysis in 1 M H_2_SO_4_. Observed polarisation curves as a function of the concentration of the catalyst: **a** [Mo_2_O_2_(*µ*-S)_2_(S_2_)_2_]^2–^; **b** [Mo_2_O_2_(*µ*-S)_2_(S_2_)(S_4_)]^2–^; **c** [W_2_O_2_(*µ*-S)_2_(S_2_)(S_4_)]^2–^; and **d** corresponding Tafel plots

To investigate the HER activity of the three clusters we chose a glassy carbon electrode (GCE) as the support. This allowed us to prepare a wide range of loading concentrations on the electrode surface by a simple drop-casting technique. In doing so we were able to examine the effects of catalyst loading on the observed HER activity, as well as determine the highest performing ratio of components used to prepare the catalytically active ink (Supplementary Table 1). The results presented here have been normalised to the geometric area coverage of the material on the electrode, and all potentials are quoted vs. the reversible hydrogen electrode (RHE). More specifically, we investigated the electrocatalytic HER activities for our three clusters, [Mo_2_O_2_(*µ*-S)_2_(S_2_)_2_]^2–^ **1**, [Mo_2_O_2_(*µ*-S)_2_(S_2_)(S_4_)]^2–^ **2** and [W_2_O_2_(*µ*-S)_2_(S_2_)(S_4_)]^2–^ **3** in 1 M H_2_SO_4_ using a typical three electrode set-up (Supplementary Figure 10). The scan rate for all linear sweep voltammetry (LSV) tests was 5 mV s^–1^, and the obtained polarisation curves were iR corrected to compensate for any potential loss arising from external resistance of the electrochemical system. Various amounts of catalyst were deposited on the GCE and formed stable thin layer film. To examine the effects of catalyst loading, the amount of catalyst on the electrode surface was controlled by adjusting the loading concentration and volume. The performance of the catalyst improves as a function of the catalyst loading up to the value of 2.85 μmol cm^−2^. Beyond this value the quality and mechanical stability of the produced electrocatalytic film is poor which leads either to similar or inferior performance due to detachment of the film from the electrode’s surface. The polarisation curves (*j*–*V* plots) recorded for catalysts **1**–**3** can be found in Fig. 3, respectively. The results indicate that the clusters are highly active towards HER revealing a small onset-overpotential depending on catalyst loading. Consequently, further increase of the applied overpotential results in a rapid rise of the cathodic current, which is indicative of HER activity. This is in sharp contrast to bare GCE with carbon powder which is relatively inert towards HER.

To obtain insight into the electron transfer kinetics, Tafel slopes were recorded for catalysts **1**–**3**. The linear portions of the Tafel plots were fit to the Tafel equation (*η* = *b* log *j* + *a*), where *η* is the overpotential, *j* is the current density, and *b* is the Tafel slope, and are displayed in Fig. 3. The HER pathway in acidic media is thought to proceed through three possible reaction steps (Table 1).

**Table 1:** Underlying electrochemical processes occurring during the H evolution reaction

(a)	Volmer step	H_3_O^+^ + e^–^ → H_ads_ + H_2_O	Discharge step
(b)	Heyrovsky step	H_ads_ + H_3_O^+^ + e^–^ → H_2_ + H_2_O	Electrochemical desorption step
(c)	Tafel step	H_ads_ + H_ads_ → H_2_	Recombination step

The HER requires a combination of two steps, either proceeding via the Volmer–Heyrovsky or Volmer–Tafel mechanism. Ideally, an efficient HER catalyst reaches the highest possible current density at the lowest possible overpotential. Therefore, smaller Tafel slopes are more desirable, as this allows for greater increase in current density with smaller increase in potential. Depending on the rate limiting step in the HER pathway the Tafel slope should be ~120, 40, or 30 mV dec^–1^, if the rate limiting step is (a), (b) or (c), respectively. For example, platinum is the best HER electrocatalyst and is known to proceed via the Volmer–Tafel mechanism, where the recombination step is rate limiting at low overpotentials, as attested by the measured Tafel slope of ~ 30 mV dec^–1^. In this case, the recorded Tafel slopes were found to be 52, 55, and 100 mV dec^–1^ for catalysts **1**, **2**, and **3**, respectively. The fact that the experimentally obtained Tafel slope values appear to be higher than the relevant value observed for the Pt catalytic surfaces, is indicative of an efficient Heyrovsky pathway.

The overpotential required to reach a current density of *j* = 10 mA cm^–2^ is widely viewed as an appropriate measure to compare total electrode activity. Furthermore, we sought to explore the effects of catalyst loading on the observed overpotential. Figure 3 shows that increasing the catalyst loading (0.05–2.85 µmol cm^–2^) results in a surge in HER activity for each catalyst. Inspection of the various catalyst loadings reveals that catalysts **1** and **2** both achieve an exceptionally low overpotential of –114 ± 3 and –114 ± 2 mV (*j* = 10 mA cm^–2^), respectively. Likewise, catalyst **3** also displays a substantial change of >200 mV, resulting in an overpotential of 227 ± 2 mV. The measured overpotentials observed for the reported loadings have been repeated at least five times and the average observed values are reported along their relevant standard deviations.

**Fig. 4:**
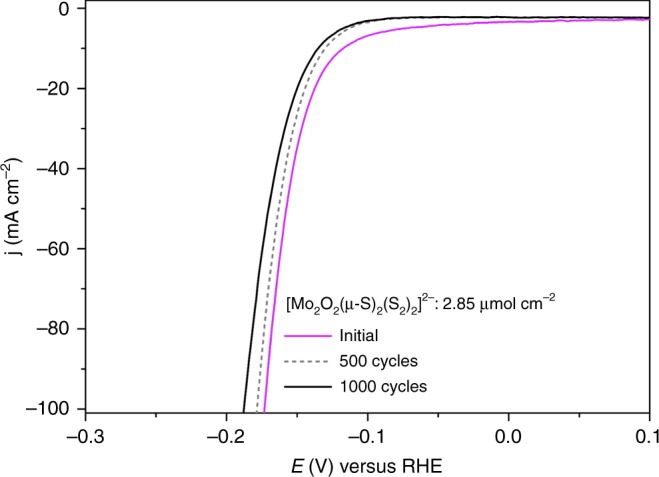
Polarisation curve of [Mo_2_O_2_(*μ*-S)_2_(S_2_)_2_]^2–^ catalyst before (magenta line) and after 1000 cycles (black line)

An important prerequisite of functional components used for application in electrochemical water-splitting devices is the high durability of the material used. In this case the stability of the molecular electrocatalysts is crucial. To assess this, we employed potential cycling as a modest simulation of the start-up/shutdown conditions found in an electrolyser to provide us with information on the catalysts’ long-term stability. Using catalyst **1** (2.85 µmol cm^–2^), we applied an accelerated scan rate of 100 mV s^–1^ between a potential range of 0.21 to −0.16 V (*V* vs. RHE) for 1000 cycles (Fig. 4). At the end of the cycling, the potential required to reach a current density of 10 mA cm^–2^ was marginally shifted by a value of <18 mV. Equally, the catalyst showed negligible loss in cathodic current density, reaching an impressive density of *j* > 100 mA cm^–2^ well below 200 mV. These results attest to the electrochemical stability of [Mo_2_O_2_(*µ*-S)_2_(S_2_)_2_]^2–^ and its potential use in electrochemical HER devices. Catalysts **2** and **3** equally showed marginal change of the observed overpotential after 1000 cycles (Supplementary Figures 11 and 12). In an effort to verify further the stability of the molecular catalysts, we decided to use three different spectroscopic methods in order to evaluate the stability of the catalyst before and after 1000 cycles. Thus, we repeated the same experiment under identical conditions using a large surface GCE (Supplementary Figure 13) in order to collect enough material for further studies. Initially, we recorded the Raman and FT-IR spectra of the three catalysts before and after the catalytic procedure (Supplementary Figures 14–16, 41–43, Supplementary Tables 14–16, Supplementary Note 1). Additionally, after the catalytic procedure we extracted the molecular species from the catalytic ink in organic solvent and investigated again the speciation in solution using high-resolution ESI-MS (Supplementary Figures 17–19). Finally, the cyclic voltammograms of the surface attached catalyst were recorded in organic medium and compared them with the CVs of freshly prepared complexes in solution (Supplementary Figures 20–22; Supplementary Note 2). All the above data show that the catalysts retain their integrity after 1000 cycles under the reported experimental conditions.

**Fig. 5:**
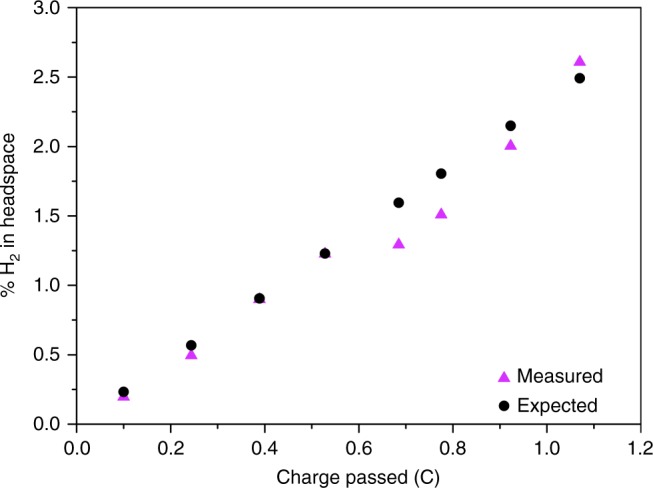
A representative trace of the gas chromatographic analysis of the single-cell headspace during the electrolysis of [Mo_2_O_2_(*μ*-S)_2_(S_2_)_2_]^2–^. The expected proportion (%) of H_2_ in the headspace was calculated from the charge passed, and the proportion (%) of H_2_ measured experimentally was determined using gas chromatography. Magenta triangles represent the actual measurements of the proportion of H_2_ in the cell headspace

Finally, to confirm that the reaction taking place on the working electrode corresponds to the HER, the evolved gas was analysed using gas chromatography (GC). As such the faradaic efficiency of **1** in 1 M H_2_SO_4_ was determined to be 92 ± 9% with a TOF of 0.12 s^−1^ at 200 mV. Figure 5 displays a representative trace of the GC analysis of the headspace in an airtight cell. Likewise, faradaic efficiencies and TOF numbers for the catalysts **2** and **3** were determined to be 90 ± 12% (TOF = 0.12 s^−1^ at 200 mV) and 93 ± 9% (TOF = 0.13 s^−1^ at 200 mV), respectively (Supplementary Figure 23). The above observation demonstrates that the catalysts evolve hydrogen at full faradaic efficiency.

### Computational studies and mechanistic insights

**Fig. 6:**
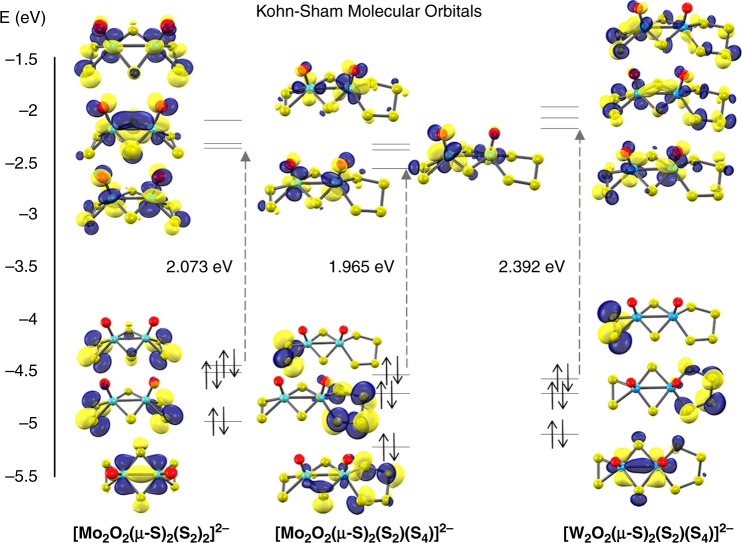
Comparative molecular orbital analysis of the oxo-sulphide anions [Mo_2_O_2_(*µ*-S)_2_(S_2_)_2_]^2–^, [Mo_2_O_2_(*µ*-S)_2_(S_2_)(S_4_)]^2–^ and [W_2_O_2_(*µ*-S)_2_(S_2_)(S_4_)]^2–^

To investigate further the underlying processes and explore potential reaction pathways, we studied the electronic structure of these molecules and their intermediate adducts based on density functional theoretical (DFT) calculations. The geometries of all the anions were optimised based on the structures obtained from the single crystal X-ray diffraction data. The electronic structure of each of these anions is quite similar and according to orbital analysis and charge distribution both the bidentate κ^1,4^-S_4_^2–^ ligand and the side-on persulfide η^2^-S_2_^2–^ formally contribute –2 charges each so the metal centres have a formal oxidation state of +5 as corroborated by the bond valence sum calculations analyses (see experimental section). Each persulfide moiety has a doubly filled π*^2^π*^2^ group molecular orbital (MO) pair. The first unoccupied MOs exhibit σ-symmetry and are predominantly metallic in character which is a characteristic feature of these species (Fig. 6). This metal bond is presented in the HOMO-3 of [Mo_2_O_2_(*µ*-S)_2_(S_2_)(S_4_)]^2–^ and HOMO-2 in the remainder anions.

Examination of the HOMO–LUMO gap provides a preliminary good estimation of the chemical potential of the species. In the present case, the tungsten-based species exhibit the highest energy separation followed by the molybdenum-based compounds according to the following order: [W_2_O_2_(*µ*-S)_2_(S_2_)(S_4_)]^2–^ > [Mo_2_O_2_(*µ*-S)_2_(S_2_)_2_]^2–^ > [Mo_2_O_2_(*µ*-S)_2_(S_2_)(S_4_)]^2–^.

### Evaluation of the hydrogen evolution process

Based on the experimental observations discussed earlier, the obtained data are indicative of an underlying Volmer–Heyrovsky process. In an effort to address the two final electrochemical steps, it is important to identify and understand the various possible pathways following the two-fold reduction of the molecular catalysts, i.e. [M_2_O_2_(*µ*-S)_2_(S_2_)(S_*x*_)]^2–^ + 2e^–^ → [M_2_O_2_(*µ*-S)_2_(S_2_)(S_*x*_)]^4–^ (M = Mo, W; *x* = 2, 4) leading to protonation equilibria. Thus, it is essential to evaluate the stability of the possible isomers formed and identify the cascade of equilibria taking place after the Volmer step. Even though these unprotonated and short-lived species may not be detected and characterised in solution, their relevant stability can be evaluated and serve as a reference state for subsequent steps. One surprising and intriguing finding obtained from the predicted geometries for these doubly reduced species is the cleavage of the persulfide bond upon 2e^–^ reduction. We showed earlier in our MO analysis (Fig. 6), that the electronic structure of these anions features metal-based orbitals which might in principle be occupied by the incoming electrons inducing the reduction of the metal centres to Mo^IV^/W^IV^. In this case, however, the existence of low lying σ*(S–S) antibonding orbitals will be preferentially occupied (Supplementary Figure 24) and consequently promote the formation of dithiolate species.

Single site protonations can take place at either oxo ligand (A), terminal persulfide site (B), or bridging sulphide position (C). The proton gain reaction was balanced using the Zundel^33^ cation (H_5_O_2_^+^) and elimination of two water molecules. The computational protocol employed in this case, involved optimising the protonated oxidised isomers H_*m*_[M_2_O_2_(*µ*-S)_2_(S_2_)(S_*x*_)]^(*m*-2)–^ followed by the addition of two unit charges on the obtained structures and re-optimise.

Supplementary Figure 25 summarises the main observations revealed by our calculations with proposed structures of the relevant intermediates. The complete nomenclature of the obtained isomers and proton affinities are listed in the Supplementary Figures 28–40. Among the isomers listed in the first tier of Supplementary Figure 25, the B1 intermediate is the most stable, since it stabilises the mitigation of the charge developed in the newly formed dithiolate species. The presence of the terminal oxo group is an important stabilisation factor in this case. The electronegativity of the oxygen centre, along with the ability to form π-bonds, contributes to the dissipation of the negative charge density developed on the terminal sulphur centres along the S∙∙∙M∙∙∙O backbone. Consequently, the second protonation step which derives from the B1 intermediate is the BB1 which exhibits a free energy value of –41.3 kcal mol^–1^. It is worth noting at this point the structural features of the formed catalyst–substrate intermediate complex BB1. Taking into consideration the Tafel slope of 52 mV dec^–1^ (see experimental discussion above) as well as the proximity of the neighbouring hydrogen atoms in this structure, raised the possibility of the existence of a Tafel-type re-combination step (Supplementary Figure 26). Further examination of this potential step for **1**–**3** (Supplementary Tables 3–13) revealed activation energies too high to be operative at room temperature and these are discussed in detail in the Supplementary Note 3.

In a similar manner, we aimed to examine the Heyrovsky step departing from the most stable H_2_[M_2_O_2_(*µ*-S)_2_(S_2_)(S_2_)_*x*_]^2–^ (M = Mo, W; *x* = 1, 2) structure as it is laid out in Table 2. This step is thermodynamically favourable across the range of molecular systems where the calculated released energies fall within the range of –8 to –9 kcal mol^–1^. In the case of the H_2_[Mo^IV^_2_O_2_(*µ*-S)_2_(S_2_)(S_4_)]^2–^ system the lowest energy isomers BC1, BC2 and BB1 were all taken into consideration since they are energetically degenerate. Alternative Heyrovsky routes can also be considered departing from the one electron reduced catalyst engaging with a solvated hydrogen atom (H·). These results are listed in Supplementary Figure 25.

**Table 2:** Heyrovsky steps departing from the most stable doubly protonated isomers

Catalytic species	Reactant complex	Δ*G*^o^ (kcal mol^−1^)
H_2_[Mo_2_O_2_(*µ*-S)_2_(S_2_)_2_]^2–^ + H_5_O_2_^+^ → H[Mo_2_O_2_(*µ*-S)_2_(S_2_)_2_]^–^ + 2H_2_O + H_2_	BB1	−7.3
H_2_[W_2_O_2_(*µ*-S)_2_(S_2_)(S_4_)]^2–^ + H_5_O_2_^+^ → H[W_2_O_2_(*µ*-S)_2_(S_2_)(S_4_)]^–^ + 2H_2_O + H_2_	BB3	−8.9
	BC1	−8.0
H_2_[Mo_2_O_2_(*µ*-S)_2_(S_2_)(S_4_)]^2–^ + H_5_O_2_^+^ → H[Mo_2_O_2_(*µ*-S)_2_(S_2_)(S_4_)]^–^ + 2H_2_O + H_2_	BC2	−7.8
	BB1	−7.7

The above experimental observations obtained from the Tafel slopes, the spectroscopic data showing the formation/elimination of S∙∙∙H/S∙∙∙S bonds, combined with the theoretical studies, provide sufficient evidence that the effective mechanistic step in the HER cycle for the specific system is the Heyrovsky one (Supplementary Note 4). This conclusion was made based on its thermodynamic favourability and the high activation energies observed for the alternative Tafel step. In the case of the Heyrovsky recombination step, the enforced proximity of the hydrogen atoms and the effective dissipation of negative charge density due to the electronegativity of the oxo centres, contributes constructively to the efficiency of the entire process which is reflected by the obtained low overpotential (–114 ± 3 mV). Subsequently, we performed an exploratory survey of the Heyrovsky mechanism occurring in the case of compound **1**. This rendered a promising candidate for the transition state which is more favourable than that of the Tafel mechanism, but the barrier height value is still overestimated (Supplementary Figure 27) with respect to the experimental one. The reason for this lack of agreement is that the proton environment is not the appropriate one. Solving this mechanistic description is a highly challenging task and will likely require multi-scale non-static approaches to gain a fuller picture of the proton transit in solution.

**Fig. 7:**
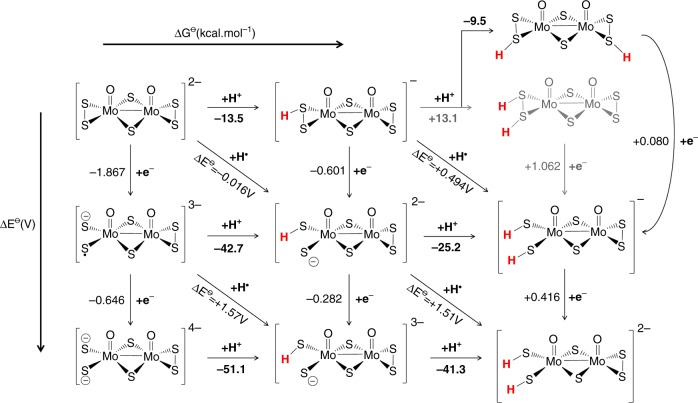
Thermodynamic routes of compound [Mo_2_O_2_(*µ*-S)_2_(S_2_)_2_]^2–^ with two variables: electron addition (vertical) and proton addition (horizontal)

Considering the symmetric structure of the [Mo_2_O_2_(*µ*-S)_2_(S_2_)_2_]^2–^ complex, we explored a series of isomers that can be formed during the two-variable proton/electron addition leading to the global panorama of possible HER equilibria (Fig. 7). In the absence of added electrons, the lowest energy structure is the neutral H_2_[Mo_2_O_2_(*µ*-S)_2_(S_2_)_2_] molecule in which two hydrogens are each bound to distant per-thiolate groups. However, upon reduction (1e^–^/2e^–^) the most stable form of the di-protonated oxysulfide is the one in which both hydrogen atoms are located at neighbouring locations. This means that upon reduction either a proton-coupled electron transfer (PCET) must take place, i.e. a proton must be removed from one of the sulphur sites in tandem with a proton–electron addition on the opposite site (diagonally in reaction scheme), or a stepwise e^−^/H^+^ addition (down and then right in Fig. 7) will take place.

It is also evident that by following a Heyrovsky-type mechanism the H[Mo_2_O_2_(*µ*-S)_2_(S_2_)_2_]^–^ (0 e^–^) anion could also be an intermediate in the catalytic cycle if a dissociative step is followed by the release of H_2_ gas. The calculated reduction potentials of one and two electron additions are low in the relevant two proton column: +0.08 and +0.416 V thus fulfilling the requirement of reversibility under the assumption that protons are easily replenished on the catalytic species.

In summary, we reported a modular approach for tuning the catalytic properties of molecular MoS_2_ edge-site mimics for the HER. The employed design principles offer the opportunity to bridge the gap between molecular and solid-state heterogeneous catalysts^34^ when immobilised on a carbon electrode surface. These molecular species represent an exciting class of low-cost earth-abundant materials and display great promise as HER electrocatalysts. Here, we prepared [Mo_2_O_2_(*µ*-S)_2_(S_2_)_2_]^2–^_,_ [Mo_2_O_2_(*µ*-S)_2_(S_2_)(S_4_)]^2–^ and [W_2_O_2_(*µ*-S)_2_(S_2_)(S_4_)]^2–^ by wet-chemical methods and investigated their HER electroactivity. Both molybdenum-based catalysts were shown to be highly active towards the HER and exhibited small Tafel slopes in the rage of ~52–55 mV dec^–1^. Notably, **1** and **2** were shown to be exceptionally active towards the HER, with a reported overpotential of just –114 ± 3 mV (at *j* = 10 mA cm^**–**2^). In addition, the excellent long-term stability of the catalyst and a remarkably high cathodic current density of 100 mA cm^–2^ at an overpotential of just 175 mV, makes these very promising candidates for future energy application in electrochemical water-splitting devices.

Additionally, we conducted electronic structural analysis and propose mechanistic pathways for the synthesised molecular catalysts by DFT calculations. The calculated HER protonation equilibria suggest a reversible cleavage and reformation of S–S bonds assisted by metal-induced polarisation. Based on the most energetically favourable calculated structures, the cleft bonds are located in the super-sulphide dihaptic ligand for H_2_[Mo_2_O_2_(*µ*-S)_2_(S_2_)_2_]^2–^ and H_2_[Mo_2_O_2_(*µ*-S)_2_(S_2_)(S_4_)]^2–^ whereas for H_2_[W_2_O_2_(*µ*-S)_2_(S_2_)(S_4_)]^2–^ they are located in the four membered chelate. Most importantly, the incorporation of the terminal oxo group seems to mitigate the negative charge density developed on the terminal sulphur centres due to its electronegativity and ability to form π-bonds. Based on the above observations, we explored both mechanistic pathways. The calculated free energies for the Heyrovsky mechanism in combination with the experimentally obtained Tafel slopes and spectroscopic data point towards the direction that Heyrovsky is the most likely pathway to be followed in the HER catalytic cycle with the exergonic steps for every catalyst ranging from –7 to – 9 kcal mol^–1^. The DFT calculations suggest that the reduction process follow the sequence H_2_[Mo_2_O_2_(*µ*-S)_2_(S_2_)_2_]^0^ + e^–^ → H_2_[Mo_2_O_2_(*µ*-S)_2_(S_2_)_2_]^–^ + e^–^ → H_2_[Mo_2_O_2_(*µ*-S)_2_(S_2_)_2_]^2–^ whereby the initial protonated species differ from that in the later reduction stages leading to the conclusion that the process must involve an electron coupled hydrogen shift in regenerating the catalyst before completing the cycle.

The results provide an elegant strategy for the preparation of a new generation of molecular catalysts exhibiting efficient HER catalytic properties. The fine stoichiometric and structural control by appropriate choice of chalcogen elements and tuning of their electronic structure, opens up the field for further discoveries and development of earth abundant molecular catalytic systems using similar design principles. With a surfeit of oxychalcogenide clusters available, this work will help us understand further mechanistic aspects of molecular analogues’ functionality and pave the way for the design of next-generation noble metal-free HER catalysts.

## Methods

### Materials and chemicals

All chemicals were purchased from Sigma Aldrich Chemical Company Ltd. and used without further purification.

### Synthesis of (NMe_4_)_2_[Mo_2_O_2_(*µ*-S)_2_(S_2_)_2_] (**1**)

The [Mo_2_O_2_(*µ*-S)_2_(S_2_)_2_]^2–^ cluster was prepared based on the method of Müller et al.^17^. In detail, a solution of (NH_4_)_2_MoO_2_S_2_ (1.8 g, 7.9 mmol) in deionized water (25 mL) was heated to boiling for about 10 min. To the above solution, NaOH (0.5 g, 12.5 mmol) and [(CH_3_)_4_N]Cl (0.8 g, 7.3 mmol) in water (75 mL), was added. Cooling the resulting solution led to the precipitation of [(CH_3_)_4_N][Mo_2_O_2_(*µ*-S)_2_(S_2_)_2_], which was recrystallised from the mother liquor, yielding orange-red crystals. The material was washed with a small amount of water, MeOH, and Et_2_O. The orange-red product was dissolved in MeCN and diffusion of Et_2_O into the MeCN solution resulted in the formation of crystals of **1** within a couple days. **1** was collected, dried and analysed. The identity and purity of the sample was confirmed by single-crystal XRD, powder XRD and FT-IR spectroscopy.

### Synthesis of (NMe_4_)_2_[Mo_2_O_2_(*µ*-S)_2_(S_2_)(S_4_)] (**2**)

The [Mo_2_O_2_(*µ*-S)_2_(S_2_)(S_4_)]^2–^ cluster was synthesised in accordance with the published procedure by Müller et al.^18^. H_2_S gas was bubbled through a suspension of sulphur (8.0 g, 249.5 mmol) in 50 mL 20% NH_3_ at room temperature. (NH_4_)_6_Mo_7_O_24_·4H_2_O (20.0 g, 16.6 mmol) was dissolved in 300 mL of boiling solution of NH_3_. The first solution was added into the second and the resultant mixture was heated with stirring at 90–95 °C for 90 min. Addition of solid [(CH_3_)_4_N]Cl (10.0 g, 91.2 mmol) to the solution gave an orange solid which was collected by filtration and washed with a minimum of cold EtOH, Et_2_O and CS_2_. The orange product was dissolved in MeCN and diffusion of Et_2_O into the MeCN solution resulted in the formation of crystals of **2** within a couple days. **2** was collected, dried and analysed. The identity and purity of the sample was confirmed by single-crystal XRD, powder XRD and FT-IR spectroscopy.

### Synthesis of (NMe_4_)_2_[W_2_O_2_(*µ*-S)_2_(S_2_)(S_4_)]·5H_2_O (**3**)

(NH_4_)_2_WS_4_ (5.0 g, 14.4 mmol) and sulphur (1.9 g, 59.3 mmol) was suspended in 70 mL DMF. The solution was degassed by bubbling N_2_, followed by heating at 110 °C for 150 min. The temperature was then lowered to 60 °C and 4 mL of water was added to the solution prior to standing for 20 h at 60 °C. The volume of the solution was reduced under vacuum at 90 °C until an oily residue was left. 20 mL of water was added to the residue and the resulting precipitate (mainly excess elemental sulphur) was removed via filtration. Ten millilitres of an aqueous solution of [(CH_3_)_4_N]Cl (3.0 g, 27.2 mmol) was added, resulting in the precipitation of an orange-yellow solid. The solid product was collected by filtration, dried with EtOH and Et_2_O. The identity and purity of the sample was confirmed by single-crystal XRD, powder XRD and FT-IR spectroscopy.

### X-ray crystallography

Data were collected at 150(2) K using a Bruker AXS Apex II [*λ*(Mo_Kα_) = 0.71073 Å] equipped with a graphite monochromator. Suitable single crystals were selected and mounted onto a rubber loop using Fomblin oil. Structure solution and refinement were carried out with SHELXS-97^35^ and SHELXL-97^36^ using the WinGX^37^ software package. Refinement was achieved by full-matrix least-squares on *F*^2^ via SHELXL-2013. Corrections for incident and diffracted beam absorption effects were applied using analytical methods. All non-hydrogen atom positions were refined anisotropically unless stated otherwise. Corrections for incident and diffracted beam absorption effects were applied using empirical absorption corrections. All the atoms were refined anisotropically. Hydrogen atom positions were calculated using standard geometric criteria and refined using a riding model. All data manipulation and presentation steps were performed using WinGX. Final unit cell data and refinement statistics for the compounds **1**–**3** are collated in Supplementary Table 2. Crystallographic data for all compounds (CCDC 1829422–1829424) can be obtained free of charge from the Cambridge Crystallographic Data Centre, 12, Union Road, Cambridge CB2 1EZ; fax:(+44) 1223–336–033, deposit@ccdc.cam.ac.uk.

### Fourier-transform infrared (FT-IR) spectroscopy

FT-IR spectra were collected in transmission mode using a JASCO FT-IR-410 spectrometer or a JASCO FT-IR 4100 spectrometer.

### Electrospray ionisation mass spectrometry

ESI-MS was performed using a Bruker micrOTOF-Q quadrupole time-of-flight mass spectrometer. Samples were dissolved in acetonitrile introduced at a dry gas temperature of 180 °C. The ion polarity for all MS scans recorded was negative, with the voltage of the capillary tip set at 4500 V, end plate offset at –500 V, funnel 1 RF at 400 Vpp and funnel 2 RF at 400 Vpp, hexapole RF at 200 Vpp, ion energy 5.0 eV, collision energy at 15 eV, collision cell RF at 1200 Vpp, transfer time at 120.0 μs, the pre-pulse storage time at 15.0 μs and analysed using the Bruker Daltonics v4.1 software.

### Powder X-ray diffraction (PXRD)

The measurements were performed using a Panalytical Xpert-pro diffractometer with CuK_α_ radiation (*λ* = 1.54178 Å) operated in Bragg–Brentano geometry.

### Raman spectroscopy

Raman spectroscopy was carried out using a Horiba Jobin–Yvon LabRam HR800 equipped with a 532 nm laser. An aperture size of 100 µm and a 1% filter was used in order to prevent sample degradation. The spectra were measured directly by focusing with a ×50 microscope on the regions of GCEs which contained the catalyst before and after cycling.

### Electrochemical measurements

The electrochemical performance was investigated using a CH Instruments CHI760D potentiostat in a custom made three-electrode electrochemical cell (Supplementary Figure 10), with 1 M H_2_SO_4_ (pH~0) as the electrolyte solution. GCE (BASi, *d* = 3.0 mm, *A* = 0.071 cm^2^) modified with different catalysts/loadings served as the working electrode, with a graphite rod as the counter electrode and a Ag│AgCl reference electrode (3 M NaCl, BASI). The electrode potentials were converted to the NHE scale by *E*(NHE) = *E*(Ag/AgCl) + 0.21 V. The polarisation curves were measured by LSV with a scan rate of 5 mV s^–1^. The measurements were recorded after degassing with Ar for 10 min. All measurements recorded have been *iR* corrected to compensate for electrolyte resistance. Tafel slopes were derived from the polarisation curves, where the logarithm of the current density is plotted against the overpotential. The accelerated stability test was applied through cyclic voltammetry at 0.21 to – 0.16 V vs. NHE with a scan rate of 100 mV s^–1^.

### Electrode preparation

The glassy carbon (GC) electrode (BASi, *d* = 3.0 mm, *A* = 0.071 cm^2^) was polished to a mirror finish with an aqueous slurry of 0.05 µm alumina powder on a nylon polishing pad (Alvatek), followed by washing with deionised water. The GC electrode was then cleaned electrochemically by cyclic voltammetry at 1 to −1.2 V for 30 cycles at a scan rate of 100 mV s^–1^. Catalysts inks were prepared in DMF. The inks consisted of a blend of catalyst/carbon powder (Cabot, Vulcan X72R) (2:1 ratio) and 5% Nafion solution (50 µL). Supplementary Table 1 summarises the exact quantities used for the investigated loadings. To form a homogenous mixture the inks were sonicated for 0.5 h. Using a micropipette, the inks were drop-cast on the GC electrode surface and dried in the oven. To examine the effects of loading concentration, various amounts of catalyst were drop-cast onto the electrode surface by control of the ink concentration and loading volume.

### Gas chromatography

GC was used to confirm that the measured currents correspond to the reduction of protons to hydrogen using an Agilent GC 7890A with a thermal conductivity detector. The GC system was calibrated using certified standards of hydrogen at various concentrations (vol%) in Ar (CK Gas Limited, UK) before use. The faradaic efficiency measurements were recorded using a single airtight cell after degassing with Ar. Galvanostatic electrolysis was performed using a two-electrode set-up that consisted of a graphite rod counter electrode, and the catalyst deposisted on a GCE as the working electrode. A constant current of –0.24 mA was applied to the system. The headspace was sampled (25 µL) and injected directly into the GC at appropriate time intervals. The faradaic efficiency was calculated by the ratio of expected H_2_ (%) in the headspace (as calculated from the charge passed) to H_2_ (%) detected using GC. Catalytic activity can also be quantified in terms of the turnover frequency (TOF). TOFs represent the number of reactant molecules converted into the desired product per unit time per catalytic site. However, specific sites may have enhanced activity compared to others, therefore the TOF is often averaged over a range of sites. Likewise, since the number of catalytically active sites in a given material is unknown, and calculations often take the entirety of the electrode material into account, the TOF is often underestimated. Nevertheless, direct comparisons of the catalytic activity can still be made provided that the estimations of TOF are carried out in a similar manner. TOF was calculated based on the amount of H_2_ evolved under galvanostatic conditions (−240 mA) over the duration of the measurement (1 h).

### Computational details

The Amsterdam Density Functional (ADF2017.1)^38^ software package was used in all the calculations. The generalised gradient density functional of Perdew, Burke and Ernzerhof^39^ with Grimme’s^40^ dispersion corrections (PBE-D3) was employed within the framework of the zeroth-order regular approximation^41,42^ (ZORA) quasi-relativistic Hamiltonian. A Slater-type orbital basis of triple zeta quality with two additional polarisation functions (TZ2P) was used for all the atoms maintaining a frozen core of 1*s*^2^ for O and S, 3*d*^10^ for Mo and 4*d*^10^ for W. Implicit solvation effects in water were added in all calculations with the COSMO^43^ scheme with default parameters. Numerical integration quality was set to ‘Good’ (six-digit precision). No symmetry constraints were imposed in the optimisations. Analytic vibrational frequencies were computed within the harmonic approximation and ideal gas conditions at 298.15 K temperature, and 1 atm of pressure. Imaginary frequency displacements were followed in the forward and backward directions by a small fraction and re-optimised to obtain products and reactants. Cationic hydrogen addition steps were balanced with the calculated free energies of the Zundel cation (H_5_O_2_^+^) and two water molecules (2H_2_O). Absolute reduction potential of half-cell reactions were obtained from the free energies with the Nernst equation E* = –Δ*G*°/(*nF*) (*F* = Faraday constant, *n* electron steps) and the values subsequently shifted relative to the normal hydrogen electrode (NHE) with the experimental absolute reduction potential^44^ E*(NHE) = – 4.24 V such that *E*° = E*_calc_ – 4.24.

## Supplementary information

Supplementary Information

Description of Additional Supplementary Files

Supplementary Data 1

Supplementary Data 2

Supplementary Data 3

## Data Availability

The datasets generated during and analysed during the current study are available from the corresponding author upon reasonable request. Crystallographic data have been deposited with the Cambridge Crystallographic Data Centre (CCDC 1829422-1829424) and may be obtained free of charge via www.ccdc.cam.ac.uk/data_request/cif. Full synthetic, analytical, crystallographic and topological analyses are given in the Supplementary Information. A data set collection of electrochemical raw data can be found in Supplementary Data 1–3. A data set collection of computational results is available in the ioChem-BD repository^45^ and can be accessed via 10.19061/iochem-bd-1-99.
