# Supplementary material for: Tuning and mechanistic insights of metal chalcogenide molecular catalysts for the hydrogen-evolution reaction
Source: Nat Commun. 2019 Jan 22;10:370. doi: 10.1038/s41467-018-08208-4 (PMC6342911; doi:10.1038/s41467-018-08208-4)
Supplement: Supplementary file 1 — Supplementary Information [file 41467_2018_8208_MOESM1_ESM.pdf]

Tuning and Mechanistic Insights of Metal Chalcogenide Molecular Catalysts for the Hydrogen-Evolution Reaction

James McAllister et. al.

Supplementary Figures

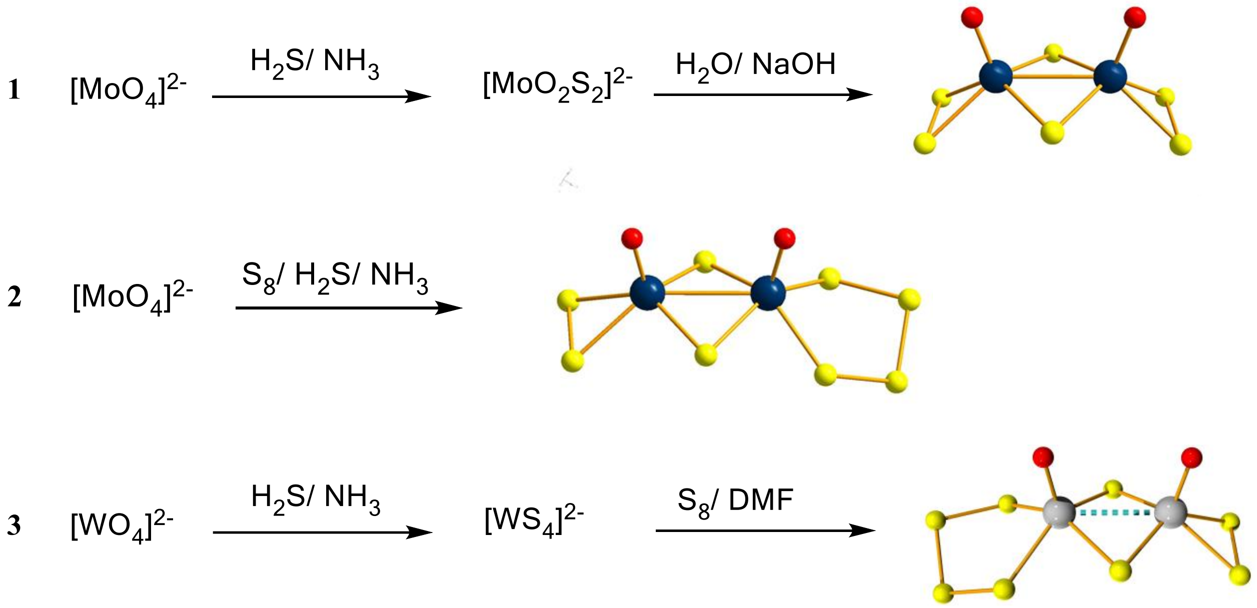

Supplementary Figure 1. Schematic showing the synthesis of catalysts $(NMe_4)_2[Mo_2O_2(\mu\text{-}S)_2(S_2)_2]$ **1**, $(NMe_4)_2[Mo_2O_2(\mu\text{-}S)_2(S_2)(S_4)]$ **2** and $(NMe_4)_2[W_2O_2(\mu\text{-}S)_2(S_2)(S_4)]$ **3**.

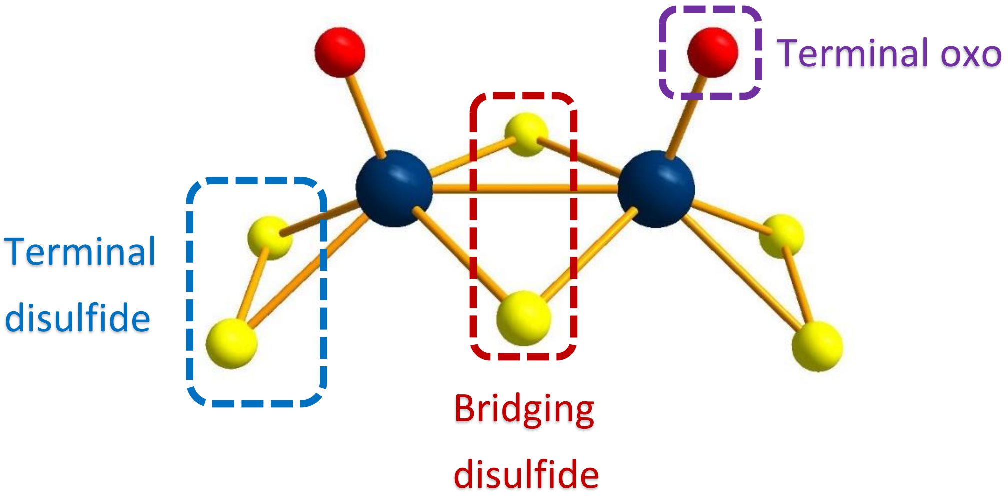

Supplementary Figure 2. Scheme of the structural features of $[Mo_2O_2(\mu\text{-}S_2)(S_2)_2]^{2-}$. The molybdenum oxo-sulfide anion possesses terminal and bridging disulfide ligands, and terminal oxo groups.

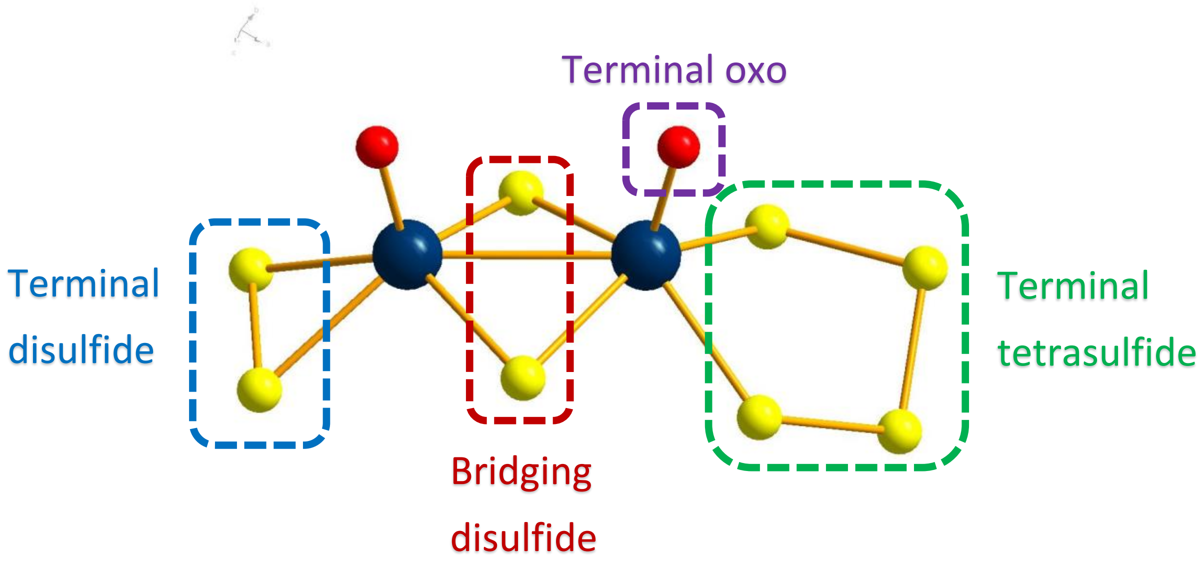

Supplementary Figure 3. Scheme of the structural features of $[Mo_2O_2(\mu\text{-}S)_2(S_2)(S_4)]^{2-}$. The molybdenum oxo-sulfide anion possesses terminal persulfide (S_2 and S_4), terminal oxo, and bridging disulfide ligands.

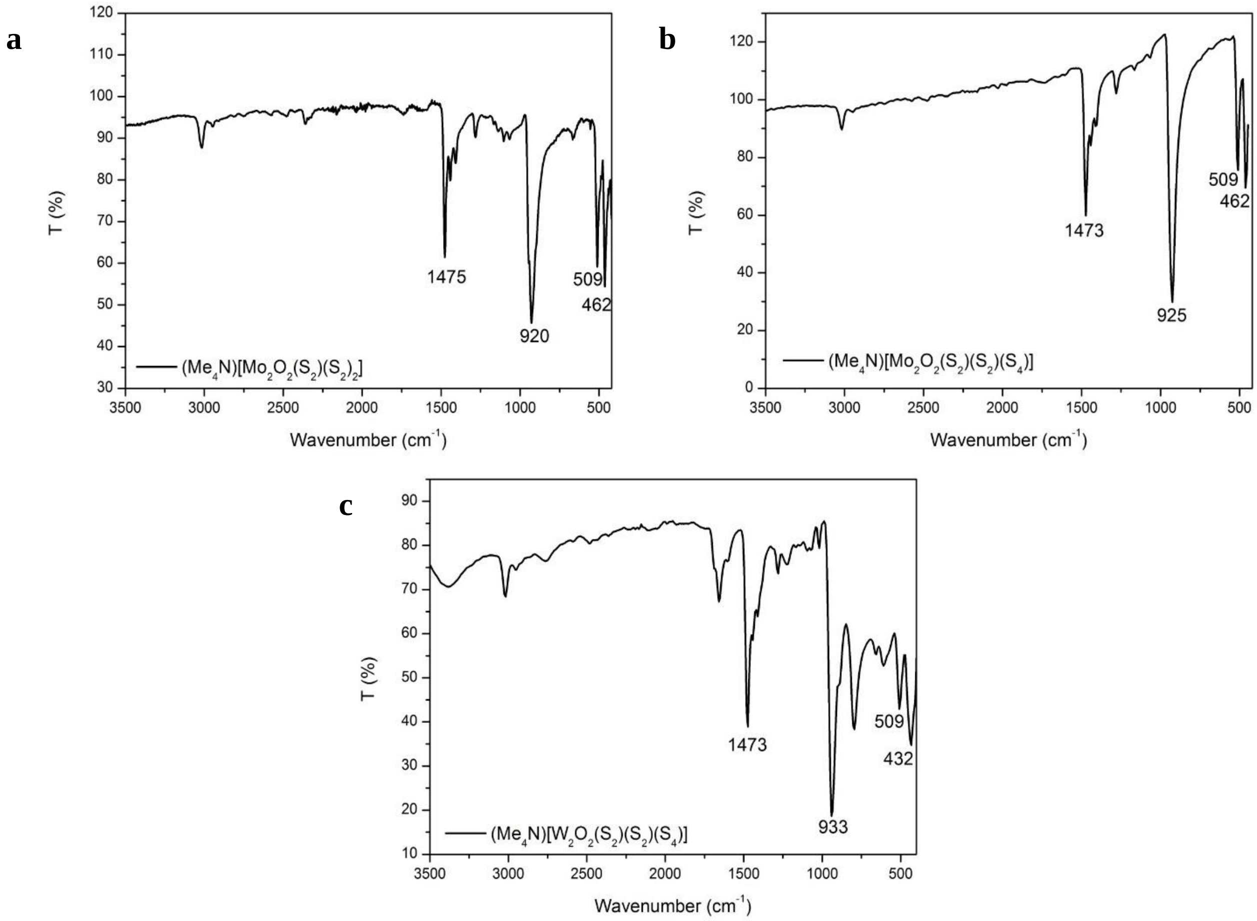

Supplementary Figure 4. FT-IR spectrum of materials. **a** $(Me_4N)[Mo_2O_2(\mu\text{-}S)_2(S_2)_2]$; **b** $(NMe_4)_2[Mo_2O_2(\mu\text{-}S)_2(S_2)(S_4)]$; **c** $(NMe_4)_2[W_2O_2(\mu\text{-}S)_2(S_2)(S_4)]$. The peaks at 1473, 1475, 1473 cm^{-1} in figures **a**, **b**, and **c** can be assigned to the vibration of NMe_4^+. The intense peak observed at 920 and 925 in **a** and **b**, is assigned to the *v*(Mo=O) vibration, and the peak at 509 cm^{-1} in both spectra is ascribed to the *v* S-S of the terminal S groups. The peak observed at 933 cm^{-1} in figure **c** is assigned to the *v* W=O vibration, and the peaks at 509 and is ascribed to the *v*(S-S) of the terminal S groups.

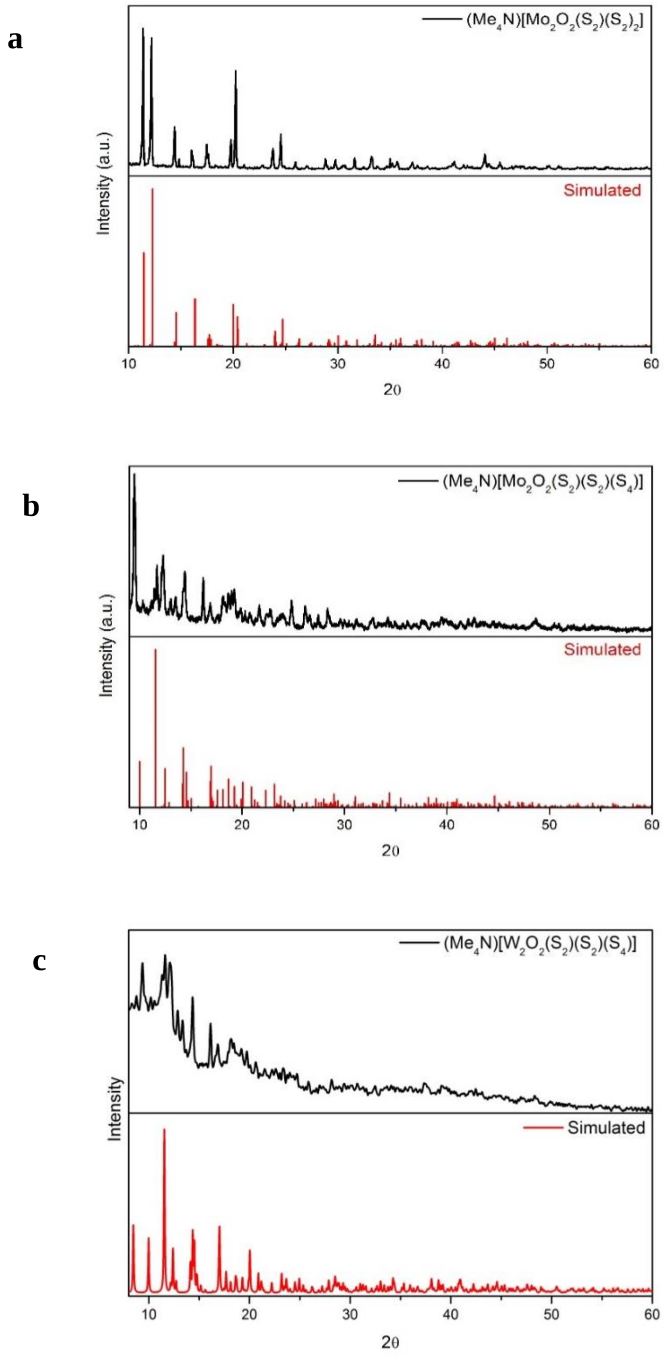

Supplementary Figure 5. Powder X-ray diffraction (XRD) pattern of **a** $(Me_4N)[Mo_2O_2(\mu\text{-}S)_2(S_2)_2]$; **b** $(NMe_4)_2[Mo_2O_2(\mu\text{-}S)_2(S_2)(S_4)]$; **c** $(NMe_4)_2[Mo_2O_2(\mu\text{-}S)_2(S_2)(S_4)]$ (black: experimental result; red: simulated based on single crystal structure)

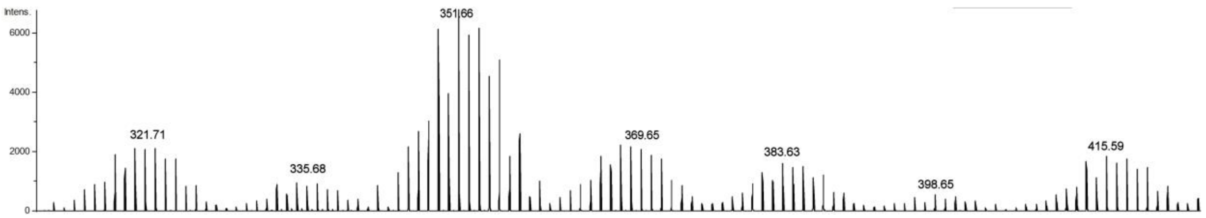

Supplementary Figure 6. ESI-MS mass spectrum of **1** (mass window 300 – 430 *m*/*z*). Other derivative species of **1** were detected during the course of the ESI-MS studies. This is due to the ionization and consecutive ion-transfer process of the charged species and has been observed previously on numerous occasions. In this case the observed envelopes centred at 415.59, 383.65, 369.69 and 351.61 m/z can be assigned to the following species: $[Mo^{V}Mo^{VI}O_2(S_2)_2(\mu\text{-}S)_2]^{-}$, $[Mo^{VI}Mo^{VI}O_2(S_2)(\mu\text{-}S)_2SH]^{-}$, $[Mo^{V}Mo^{VI}O_2(S_2)(\mu\text{-}S)_2)H_2O]^{-}$ and $[Mo^{V}Mo^{VI}O_2(S_2)(\mu\text{-}S)_2]^{-}$.

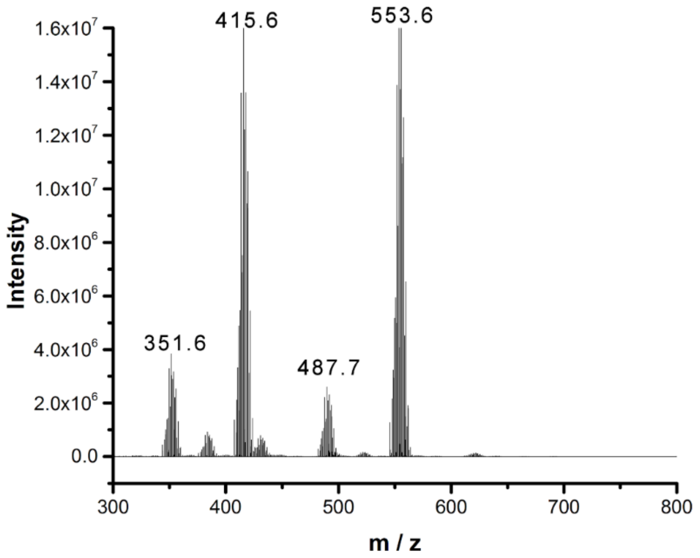

Supplementary Figure 7. ESI-MS mass spectrum of **2** (mass window 300 – 800 *m/z*). As series of derivative species of **2** were detected during the course of the ESI-MS studies. This is due to the ionization and consecutive ion-transfer process of the charged species and has been observed previously on numerous occasions. In this case the observed envelopes centred at 553.67, 415.63 and 351.61 m/z can be assigned to the following species: $\{[(CH_3)_4N][Mo^{V}_2O_2(S_2)(\mu\text{-}S)_2S_4]\}^{-}$, $[Mo^{V}Mo^{VI}O_2(S_2)(\mu\text{-}S)_2S_4]^{-}$ and $[Mo^{V}Mo^{VI}O_2(S_2)(\mu\text{-}S)_2]^{-}$.

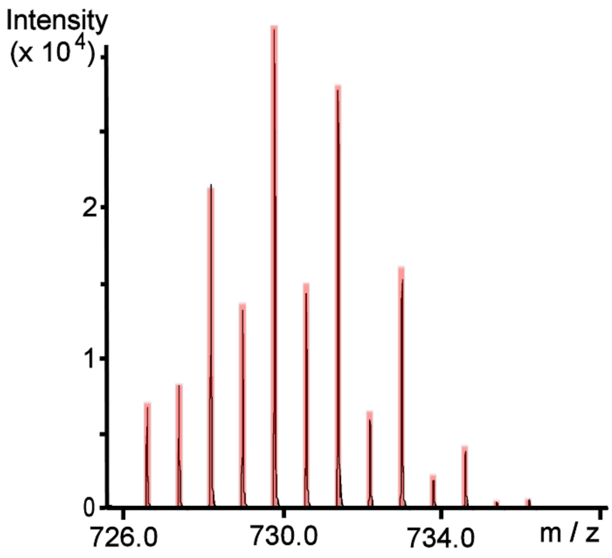

Supplementary Figure 8. Negative ion mass spectrum in CH_3CN solution of $\{[(CH_3)_4N][W_2^{VI}O_2S_8]\}^-$ **3**. The distribution envelope is centred at *m/z* ca. 729.7. Black line: experimental data, Red bars: simulation of isotope pattern.

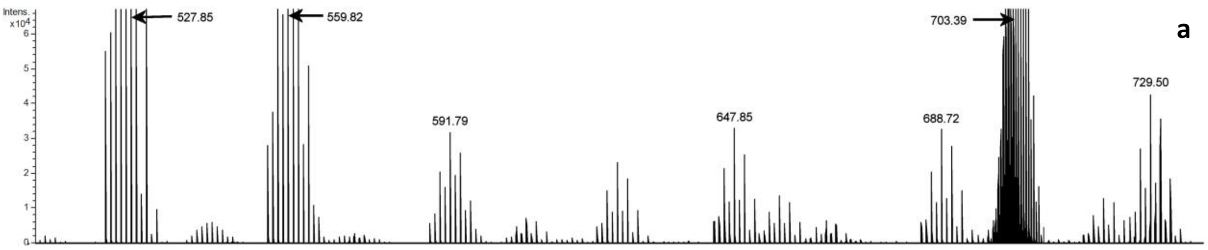

Supplementary Figure 9. ESI-MS mass spectrum of **3** (mass window 500 – 800 *m/z*). As series of derivative species of **3** were detected during the course of the ESI-MS studies. This is due to the ionization and consecutive ion-transfer process of the charged species and has been observed previously on numerous occasions. In this case the observed envelopes centred at 729.50, 703.39, 688.69, 647.85, 591.74, 559.82 and 527.85 m/z can be assigned to the following species: $\{[(CH_3)_4N][W^V{}_2O_2(S_2)(\mu\text{-}S)_2S_4]\}^-$, $[W^{IV}W^VO_2(S_2)(\mu\text{-}S)_2S_3H_2Na_2]^-$, $[W^V{}_2O_2(S_2)(\mu\text{-}S)_2S_3HNa_2\ (H_2O)]^-$, $[W^V{}_2O_2(S_2)(\mu\text{-}S)_2S_4HNa]^-$, $\{[(CH_3)_4N][W^V{}_2O_2(S_2)(\mu\text{-}S)_2S_2]\}^-$, $[W^VW^{VI}O_2(S_2)(\mu\text{-}S)_2S_2]^-$, $[W^{VI}W^{VI}O_2(S_2)(\mu\text{-}S)_2S]^-$ and $[W^{VI}W^{VI}O_2(S_2)(\mu\text{-}S)_2]^-$.

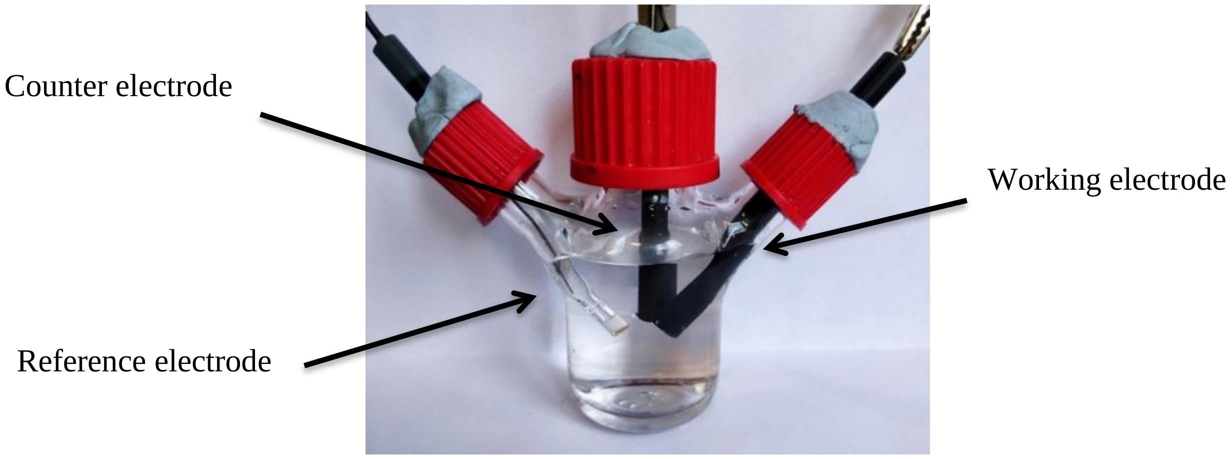

Supplementary Figure 10. Standard three-electrode electrochemical setup.

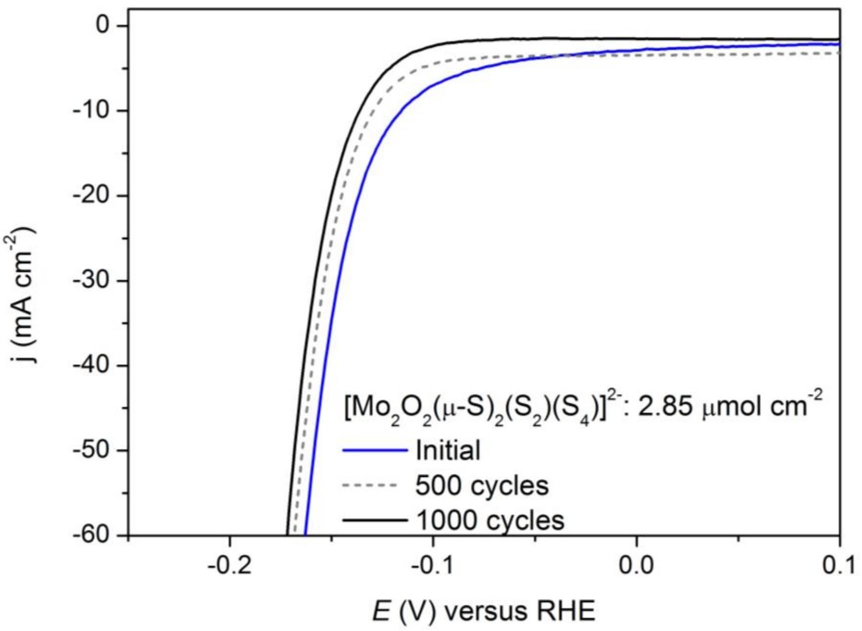

Supplementary Figure 11. Polarization curve of $[Mo_2O_2(\mu\text{-}S)_2(S_2)_4]^{2-}$ catalyst before (blue line) and after 1000 cycles (black line). A graphite rod was used as counter electrode during stability measurement.

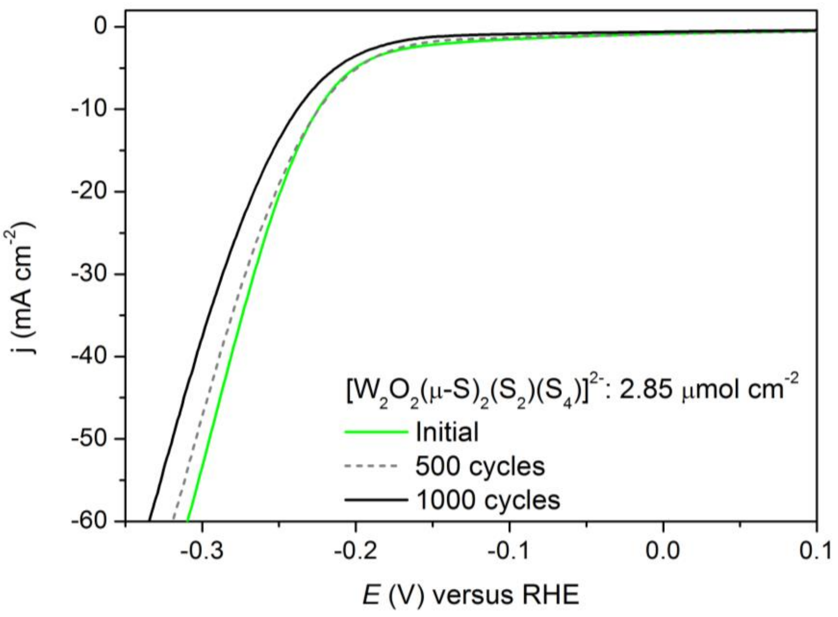

Supplementary Figure 12. Polarization curve of $[W_2O_2(\mu\text{-}S)_2(S_2)_4]^{2-}$ catalyst before (green line) and after 1000 cycles (black line). A graphite rod was used as counter electrode during stability measurement.

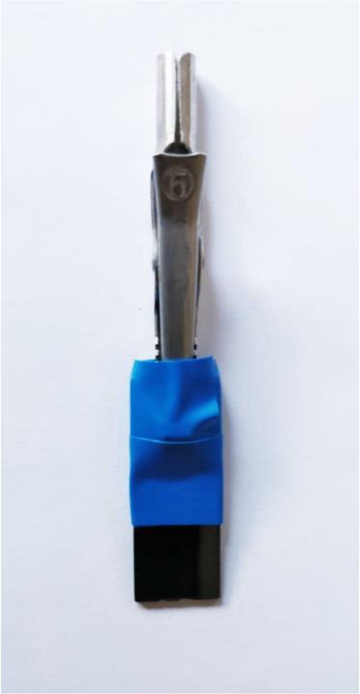

Supplementary Figure 13. Large surface glassy carbon foil used as working electrode for the stability experiments. Active surface area: 1 cm^3.

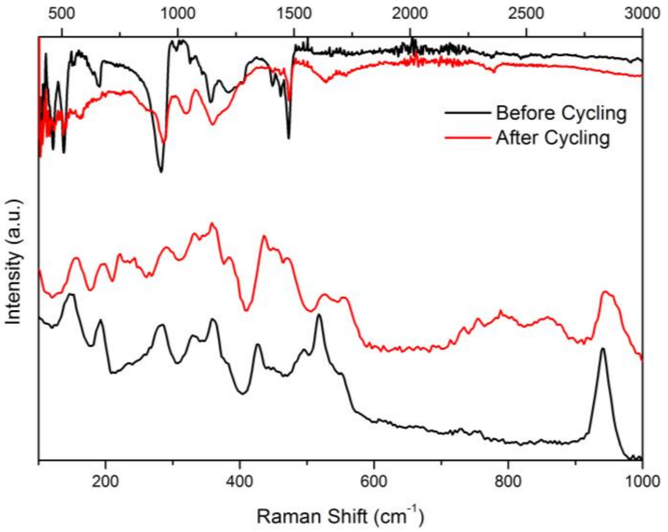

Supplementary Figure 14. Raman and FT-IR spectra of $[Mo_2O_2(\mu\text{-}S_2)(S_2)_2]^{2-}$ **1** cluster [UPPER: FT-IR spectrum of **1** on glassy carbon electrode before (black line) and after (red line) 1000 cycles; LOWER: Raman spectrum of **1** on glassy carbon electrode before (black line) and after (red line) 1000 cycles]. Fingerprint region 200-600 cm^{-1} corresponds to M-S-M and S-M-S bending vibrations while the peak located between 900-1000 cm^{-1} corresponds to the characteristic M=O stretching vibration.

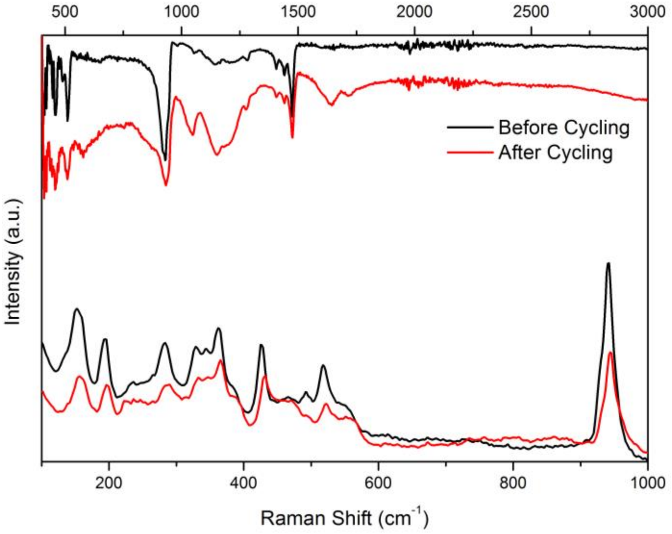

Supplementary Figure 15. Raman and FT-IR spectra of $[Mo_2O_2(\mu\text{-}S_2)(S_2)(S_4)]^{2-}$ **2** cluster [UPPER: FT-IR spectrum of freshly prepared compound **2** before (black line) and after (red line) 1000 cycles; LOWER: Raman spectrum of **2** before (black line) and after (red line) 1000 cycles]. Fingerprint region 200-600 cm^{-1} corresponds to M-S-M and S-M-S bending vibrations while the peak located between 900-1000 cm^{-1} corresponds to the characteristic M=O stretching vibration.

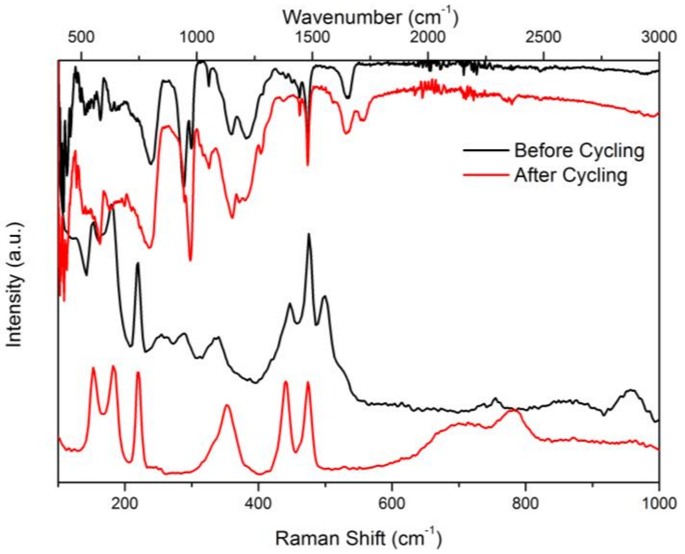

Supplementary Figure 16. Raman and FT-IR spectra of $[W_2O_2(\mu\text{-}S_2)(S_2)(S_4)]^{2-}$ **3** cluster [UPPER: FT-IR spectrum of **3** before (black line) and after (red line) 1000 cycles; LOWER: Raman spectrum of **3** before (black line) and after (red line) 1000 cycles]. Fingerprint region 200-600 cm^{-1} corresponds to M-S-M and S-M-S bending vibrations while the peak located between 900-1000 cm^{-1} corresponds to the characteristic M=O stretching vibration.

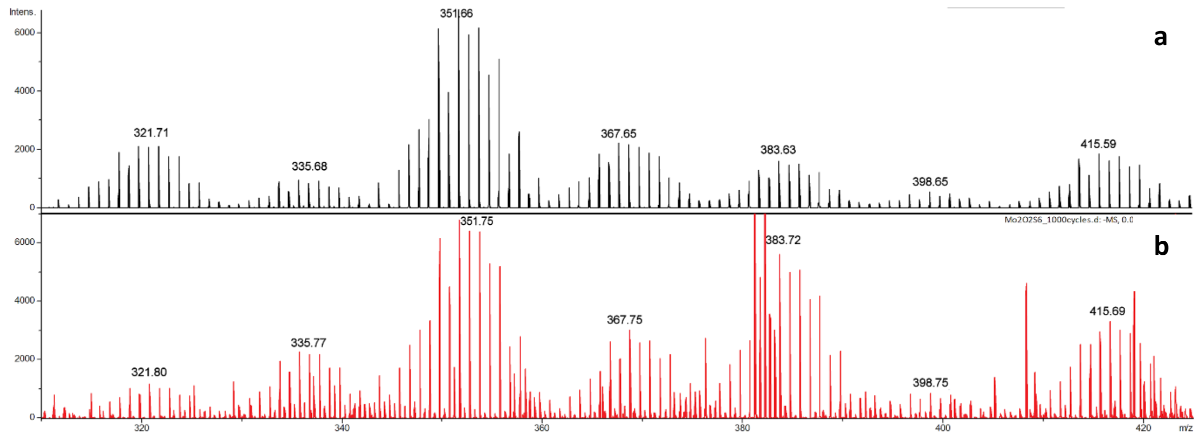

Supplementary Figure 17. ESI-MS spectrum of compound **1** before **a** and after **b** the catalytic process. The ability to extract any molecular species present after the catalytic process and the observation of the same isotopic distribution envelopes in solution confirms that the species immobilized initially on the electrode retained their structural integrity.

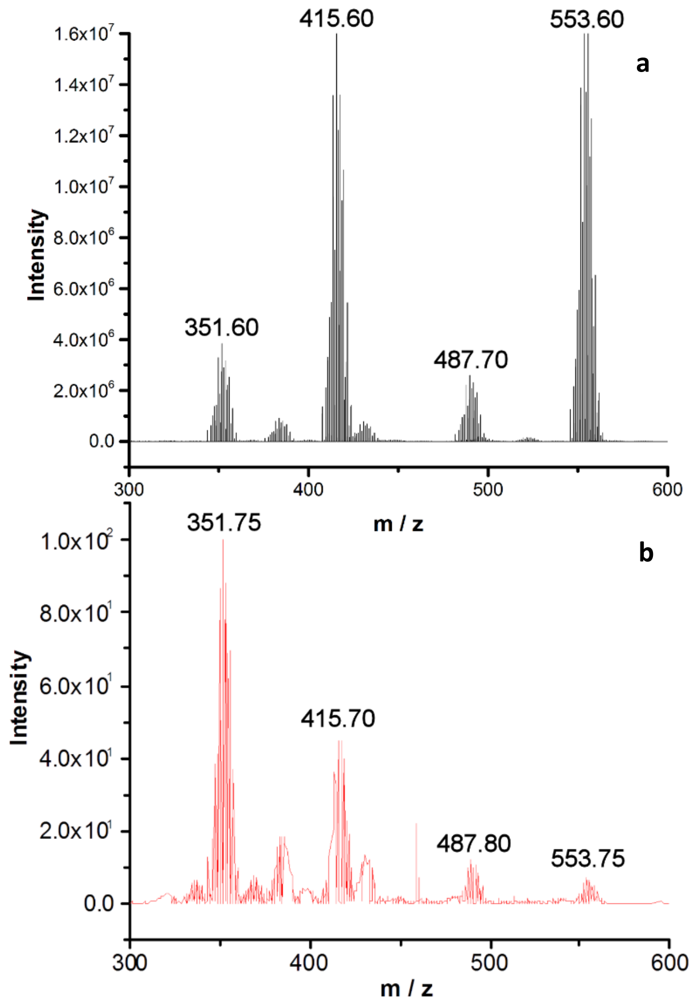

Supplementary Figure 18. ESI-MS spectrum of compound **2** before **a** and after **b** the catalytic process. The ability to extract any molecular species present after the catalytic process and the observation of the same isotopic distribution envelopes in solution confirms that the species immobilized initially on the electrode retained their structural integrity.

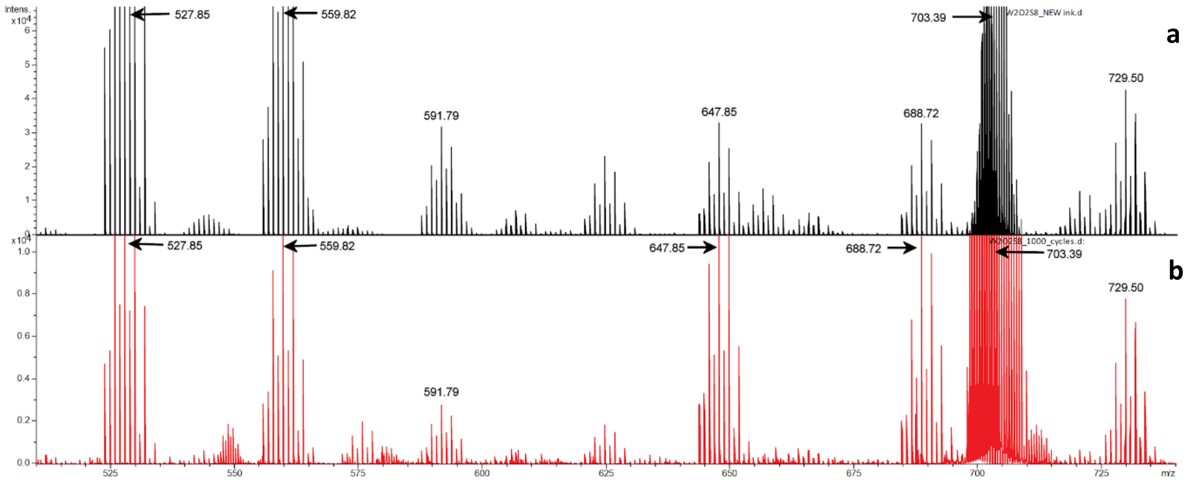

Supplementary Figure 19. ESI-MS spectrum of compound **3** before **a** and after **b** the catalytic process. The ability to extract any molecular species present after the catalytic process and the observation of the same isotopic distribution envelopes in solution confirms that the species immobilized initially on the electrode retained their structural integrity.

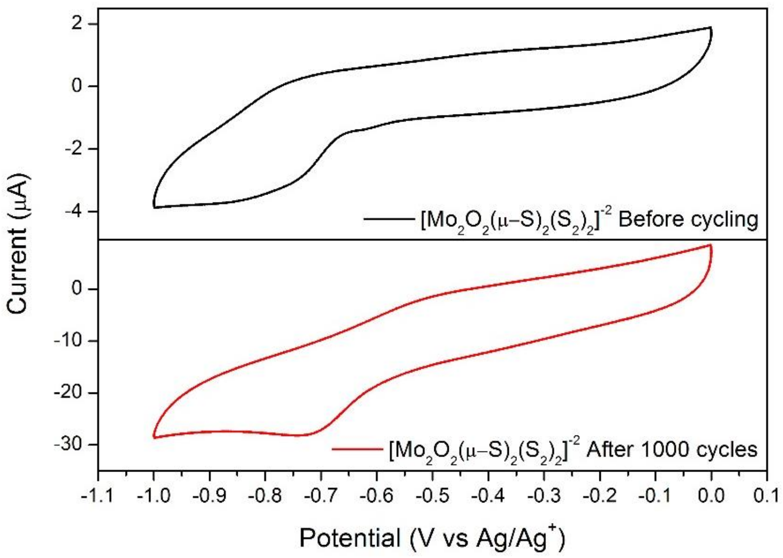

Supplementary Figure 20. Cyclic voltammograms of: catalyst **1** dissolved in dry and degassed CH_3CN/TBA-BF_4 solution (Black line); utilized catalyst **1** attached on the surface of the electrode after 1000 catalytic cycles (as described in the catalytic experimental section) and re-measured in dry and degassed CH_3CN/TBA-BF_4 (Red line).

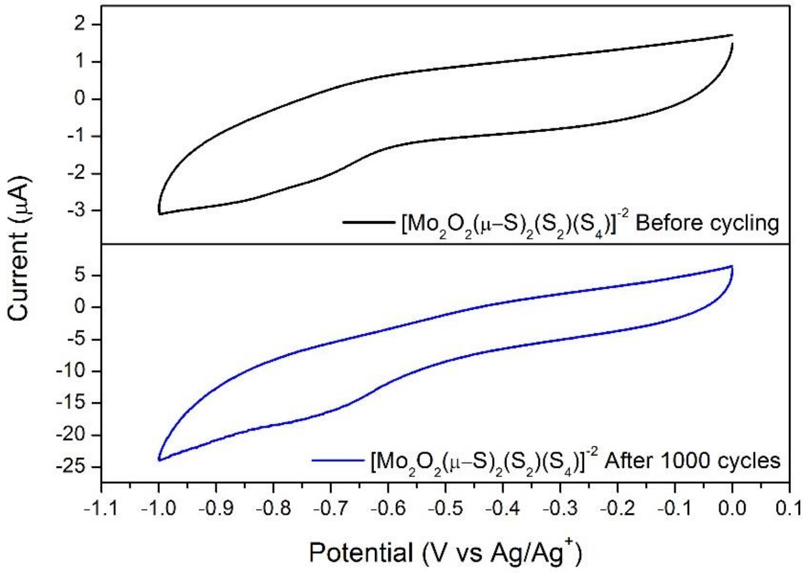

Supplementary Figure 21. Cyclic voltammograms of: catalyst **2** dissolved in dry and degassed CH_3CN/TBA-BF_4 solution (Black line); utilized catalyst **2** attached on the surface of the electrode after 1000 catalytic cycles (as described in the catalytic experimental section) and re-measured in dry and degassed CH_3CN/TBA-BF_4 (Blue line).

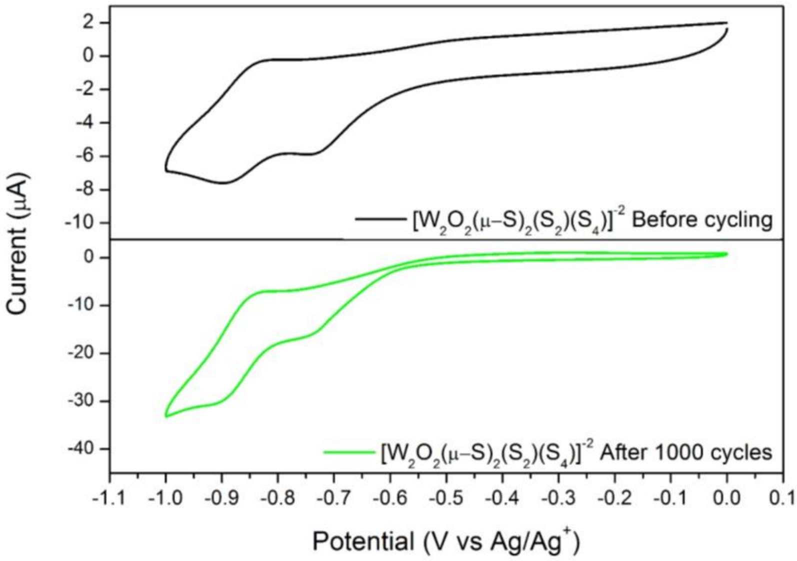

Supplementary Figure 22. Cyclic voltammograms of: catalyst **3** dissolved in dry and degassed CH_3CN/TBA-BF_4 solution (Black line); utilized catalyst **3** attached on the surface of the electrode after 1000 catalytic cycles (as described in the catalytic experimental section) and re-measured in dry and degassed CH_3CN/TBA-BF_4 (Green line).

a

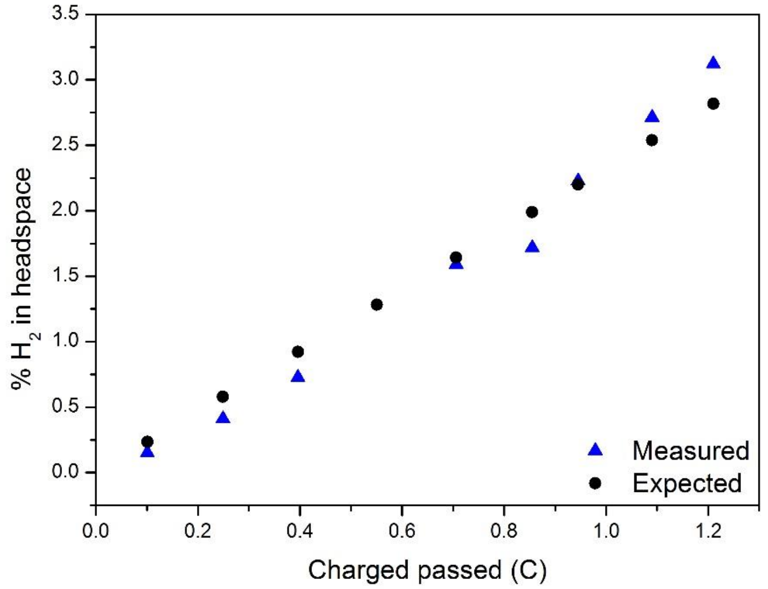

b

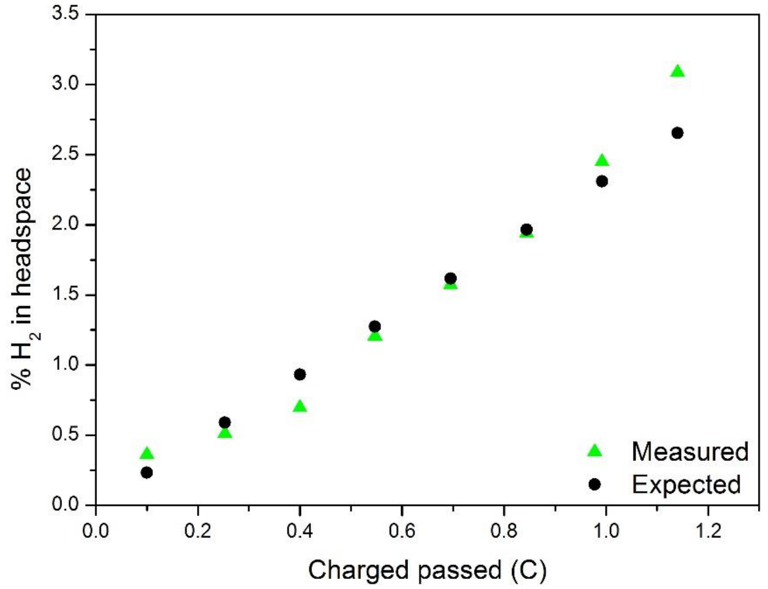

Supplementary Figure 23. Presentation of the representative trace of the gas chromatographic analysis of the single-cell headspace during the electrolysis of **a** $[Mo_2O_2(\mu\text{-}S)_2(S_2)(S_4)]^{2-}$ and **b** $[W_2O_2(\mu\text{-}S)_2\,(S_2)(S_4)]^{2-}$. The expected proportion (%) of H_2 in the headspace was calculated from the charge passed, and the proportion (%) of H_2 measured experimentally was determined using gas chromatography. Black squares represent the actual measurements of the proportion of H_2 in the cell headspace.

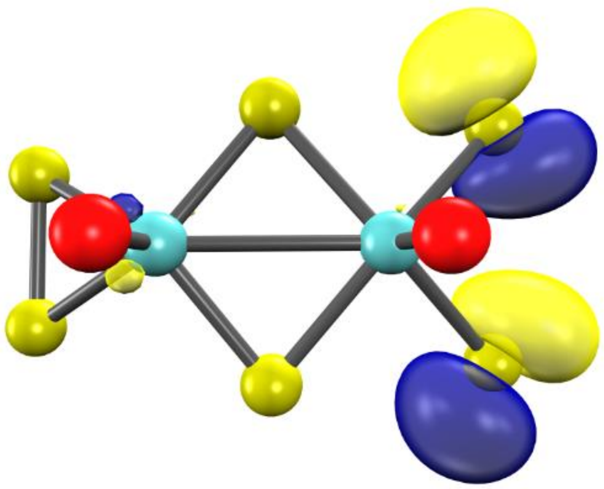

Supplementary Figure 24. HOMO of the doubly reduced $[Mo_2O_2(\mu\text{-}S)_2(S_2)_2]^{4-}$ anion.

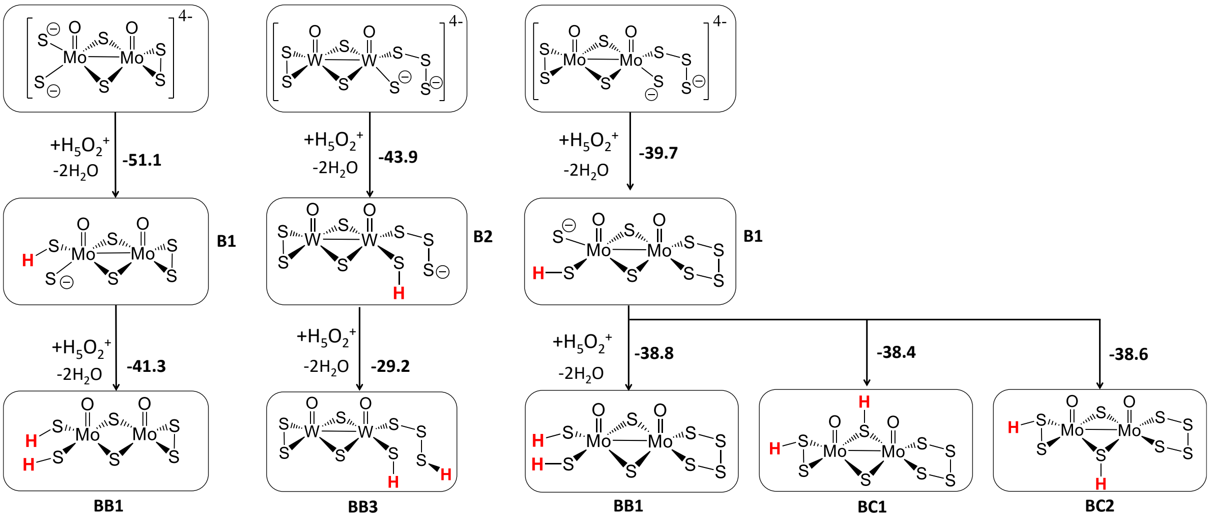

Supplementary Figure 25. Protonation equilibria of the $[M_2O_2(\mu\text{-}S)_2(S_2)(S_x)]^{4-}$ (M = Mo, W; x = 2, 4) $2e^-$ reduced species. (Free energies in kcal·mol^{-1})

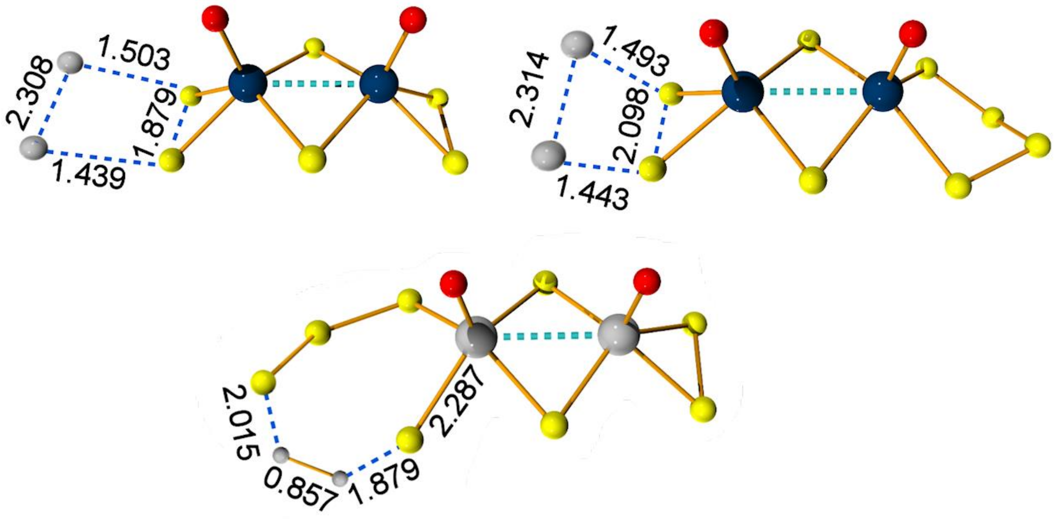

Supplementary Figure 26. Calculated Tafel transition states for the three molecular catalysts with the most relevant bond distances (Å).

Preliminary investigation of the Heyrovsky mechanism

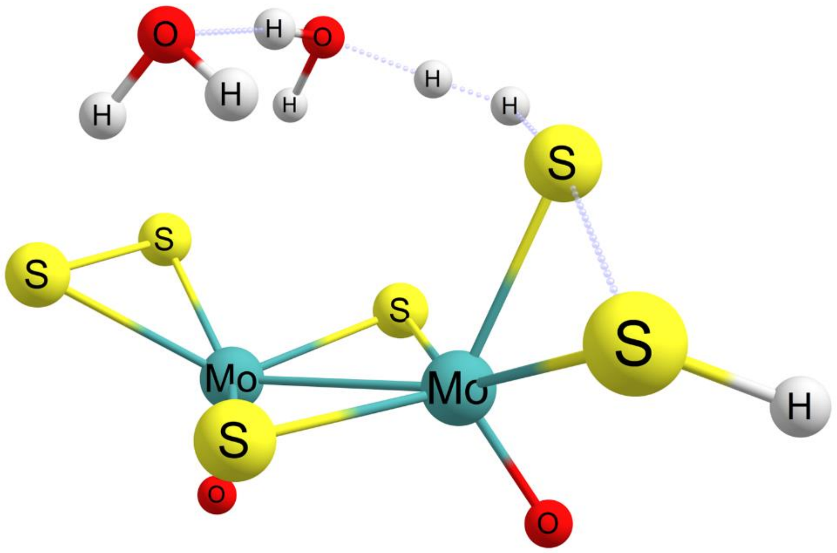

Supplementary Figure 27. Transition state model leading to molecular hydrogen release. A preliminary investigation of the external hydrogen capture by BB1 (compound **1**) was carried out by performing several constrained linear transits using the Zundel cation ($H_5O_2^+$) as the electrophilic source. The obtained transition state has an imaginary mode of 684i cm^{-1} and has a relative free energy value $\Delta G^{\ddagger}$=+33.7 kcal.mol^{-1}. It is characterised by a concerted S-S re-bonding and hydride release.

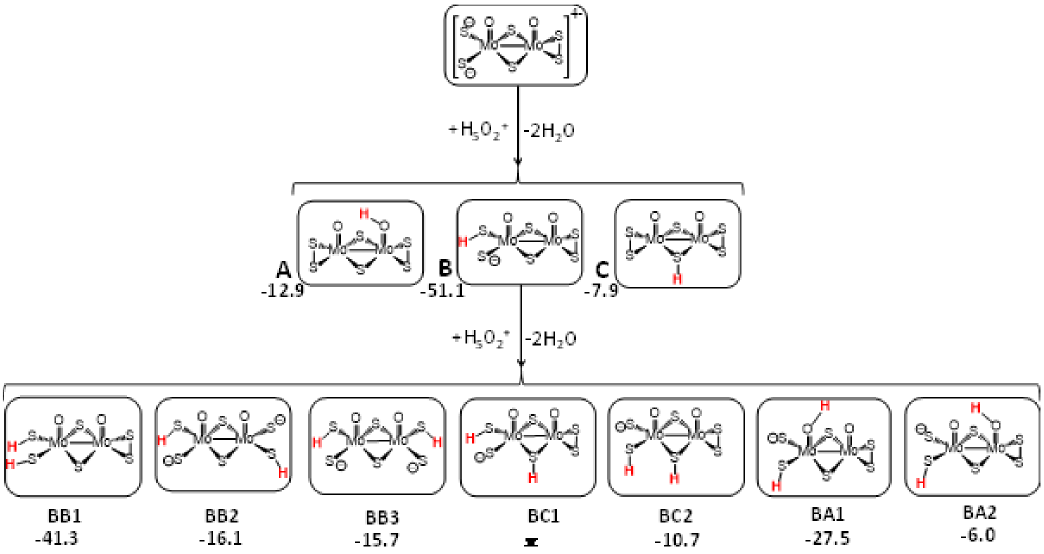

Supplementary Figure 28. Protonation genealogy of the unique (non-redundant) regio-isomers starting from the doubly reduced $[Mo_2O_2(\mu\text{-}S)_2(S_2)_2]^{4-}$ anion. Listed values are free energies in aqueous solution in kcal·mol^{-1}. Structure **BC1** could not be obtained leading instead to **BB1**.

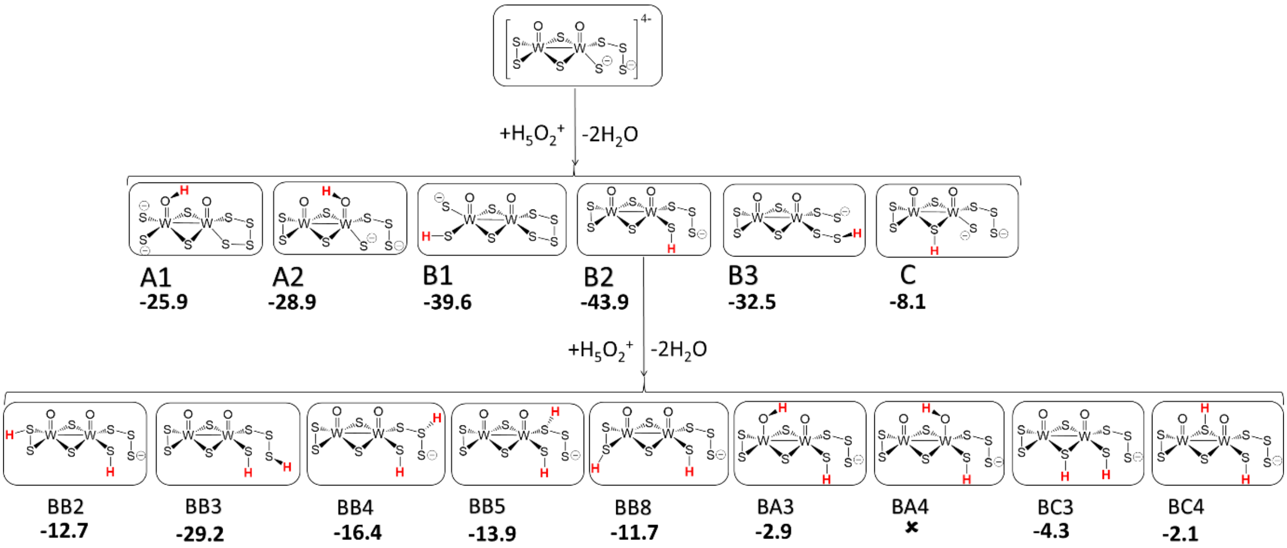

Supplementary Figure 29. Protonation genealogy of the unique (non-redundant) regio-isomers starting from the doubly reduced $[W_2O_2(\mu\text{-}S)_2(S_2)(S_4)]^{4-}$ anion. Listed values are free energies in aqueous solution in kcal·mol^{-1}. Structure **BA4** could not be obtained leading instead to **BB3**.

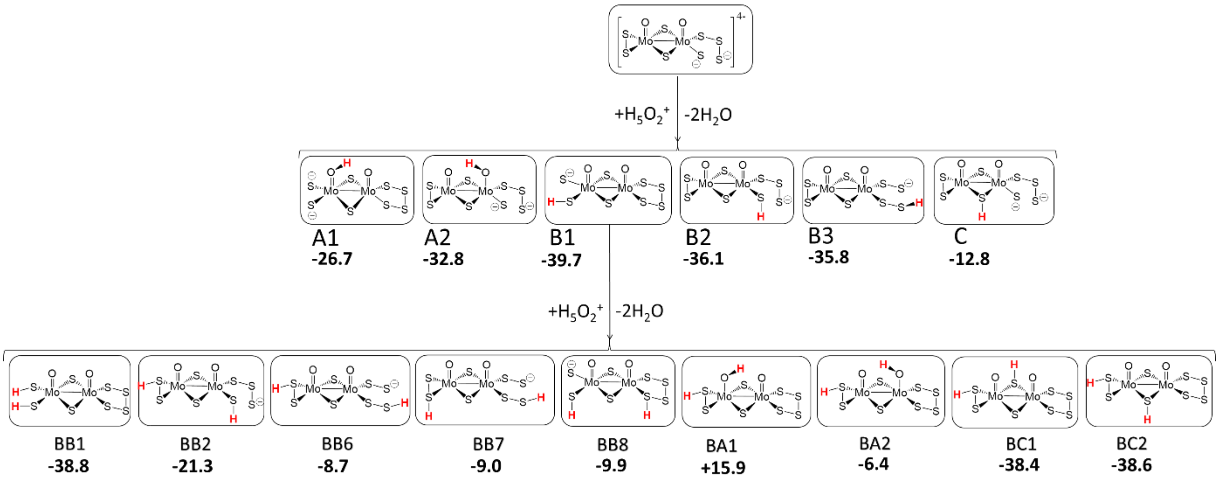

Supplementary Figure 30. Protonation genealogy of the unique (non-redundant) regio-isomers starting from the doubly reduced $[Mo_2O_2(\mu\text{-}S)_2(S_2)(S_4)]^{4-}$ anion. Listed values are free energies in aqueous solution in kcal·mol^{-1}.

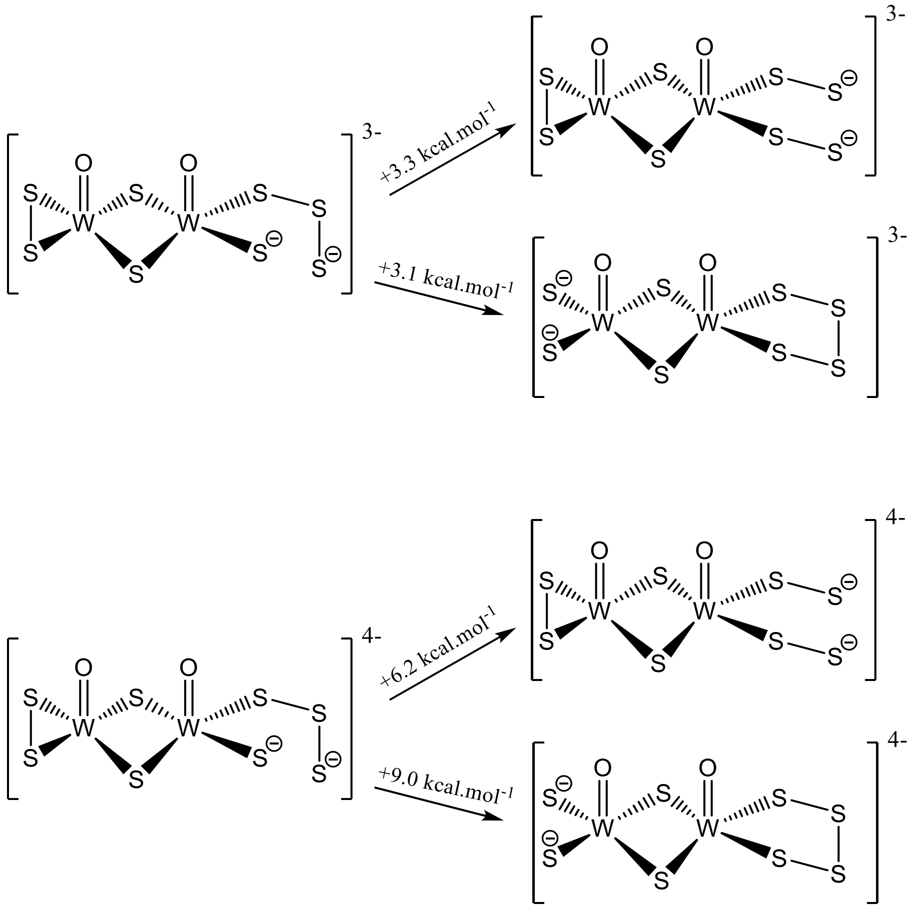

Supplementary Figure 31. Free energies of bond cleavage sites in $[W_2O_2S_8]^{3-/4-}$.

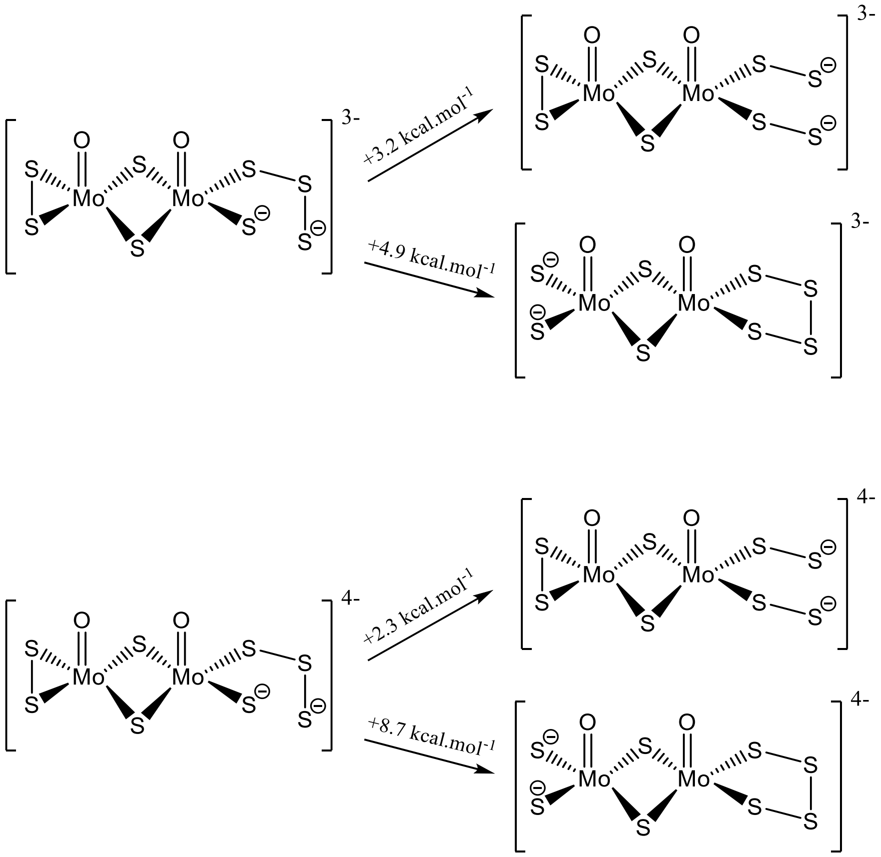

Supplementary Figure 32. Free energies of bond cleavage sites in $[Mo_2O_2S_8]^{3-/4-}$.

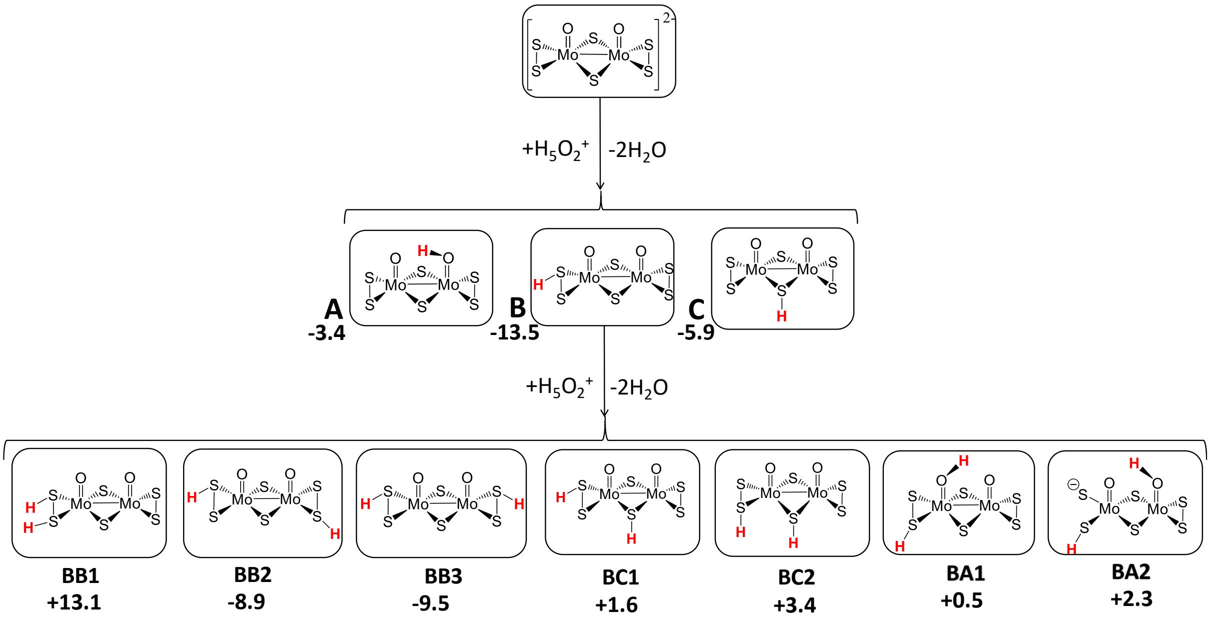

Supplementary Figure 33. Protonation genealogy of compound **1** without any added electrons.

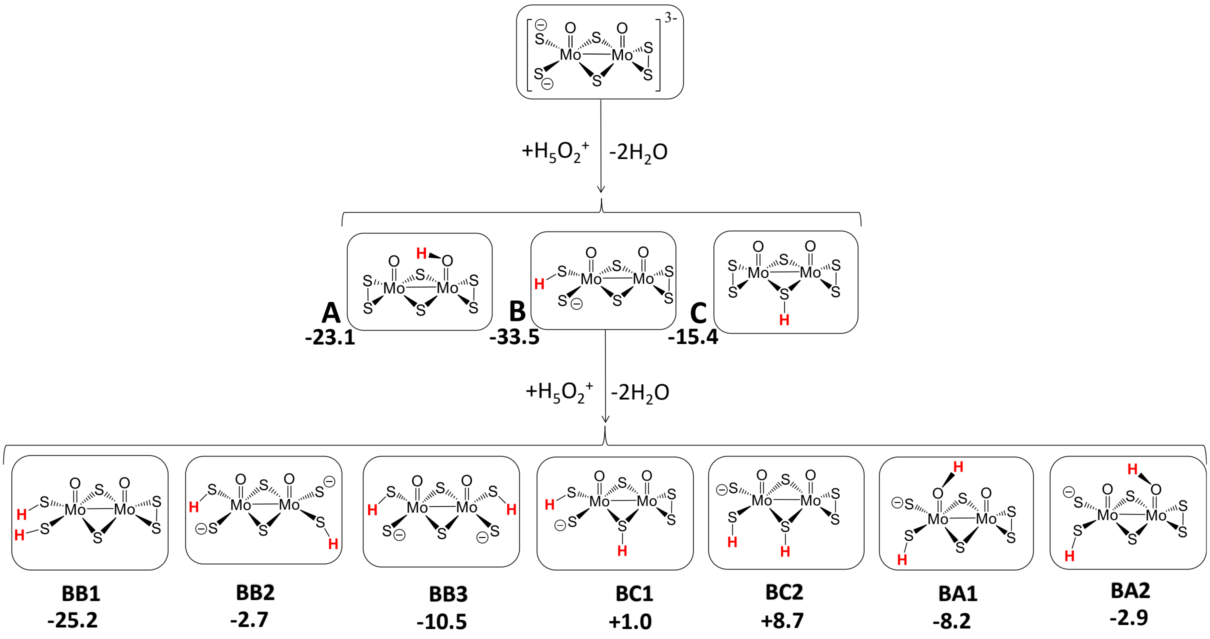

Supplementary Figure 34. Protonation genealogy of singly reduced compound **1**.

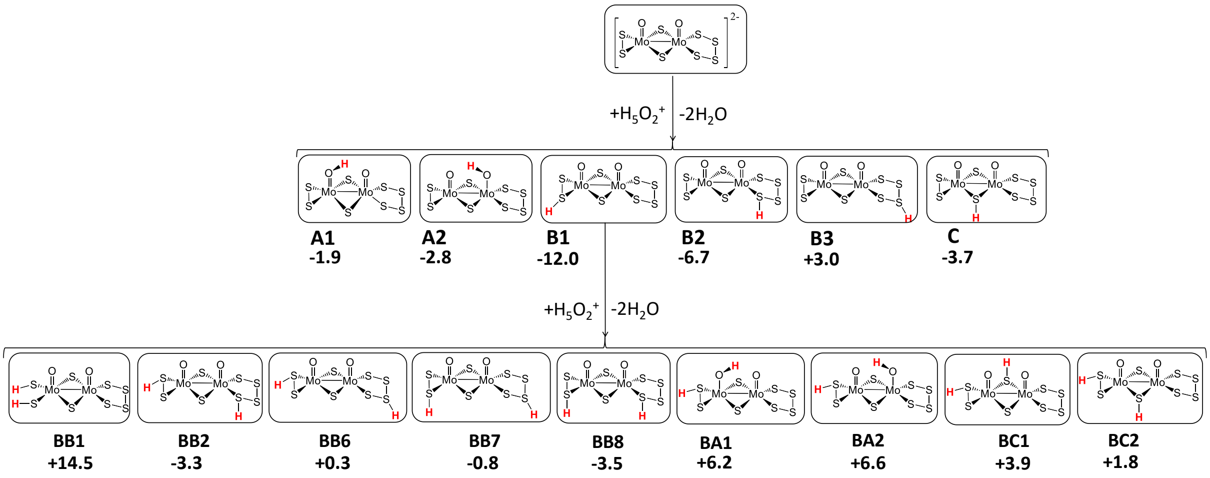

Supplementary Figure 35. Protonation genealogy of fully oxidised compound **2**.

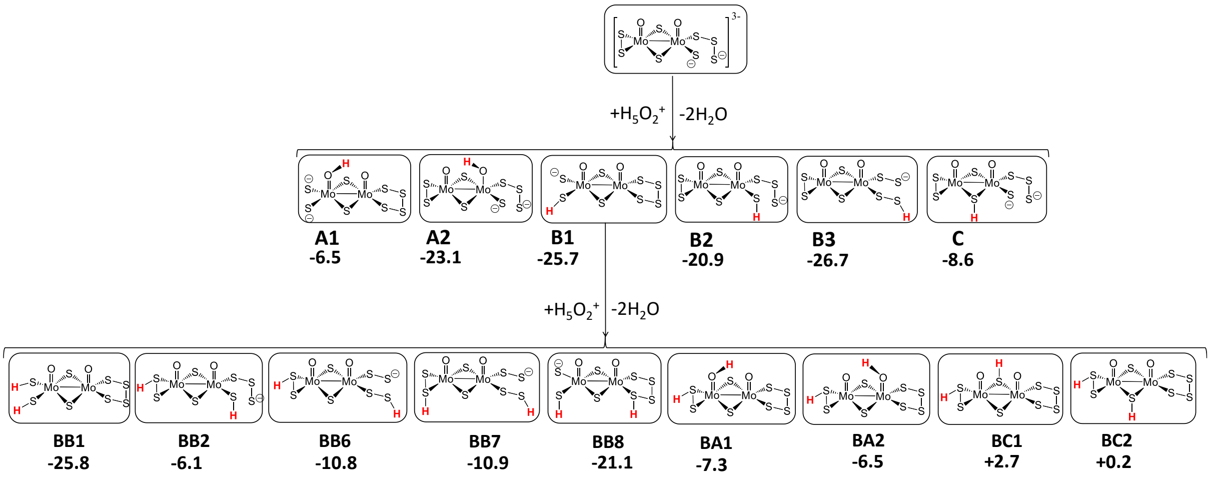

Supplementary Figure 36. Protonation genealogy of singly reduced compound **2** showing the B1 branch. Notice that B3 is nearly degenerate with B1 so this route was also explored (see below). Notwithstanding BB1 is still the most favourable structure overall.

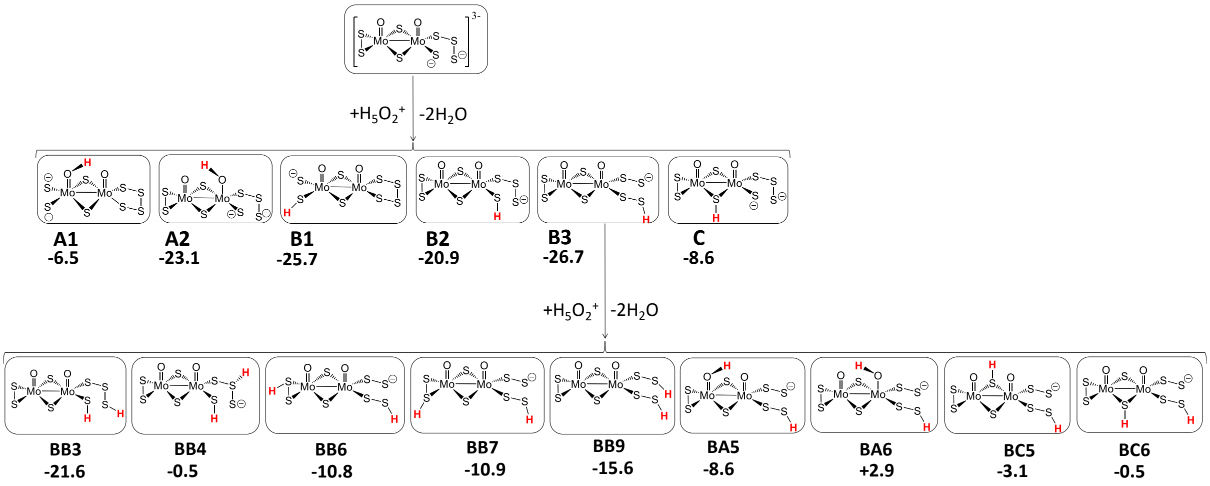

Supplementary Figure 37. Protonation genealogy of singly reduced compound **2** showing the B3 branch.

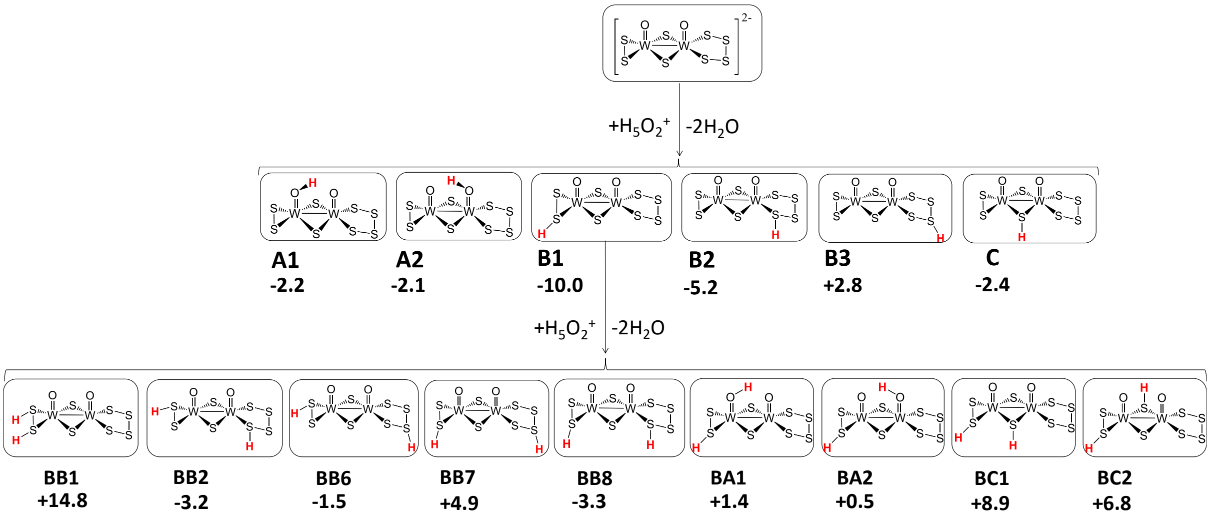

Supplementary Figure 38. Protonation genealogy of fully oxidised compound **3**.

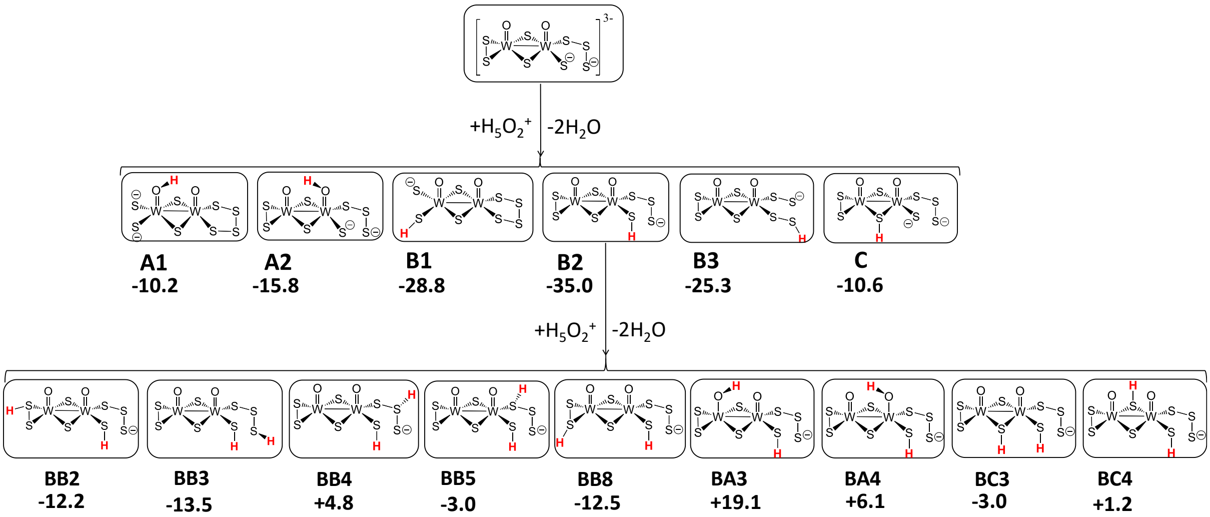

Supplementary Figure 39. Protonation genealogy of singly reduced compound **3**.

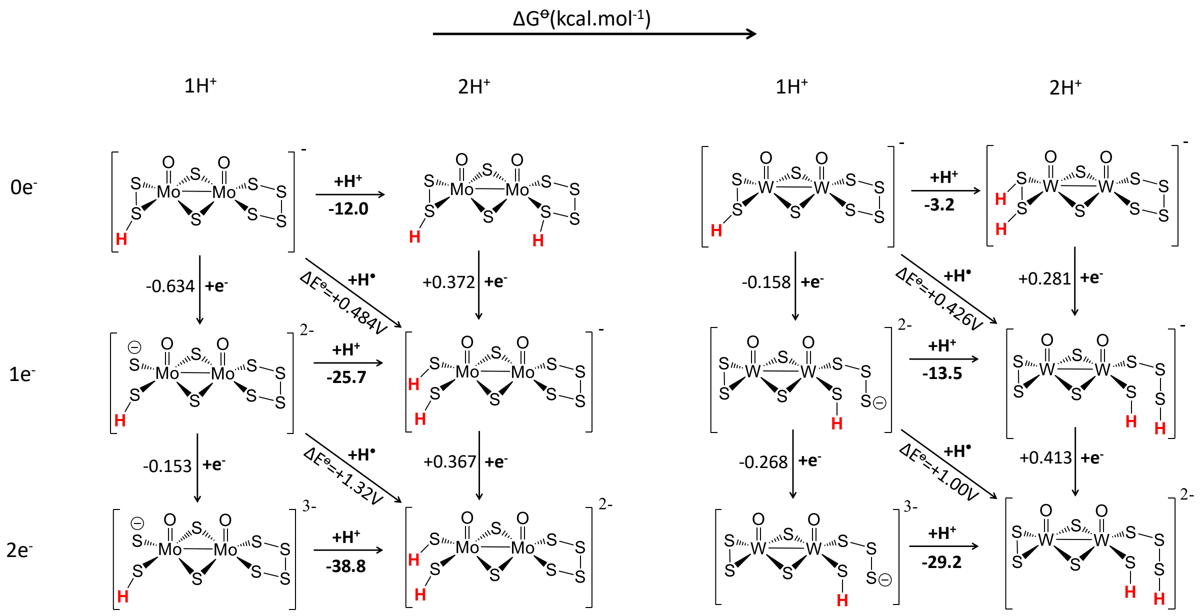

Supplementary Figure 40. Generic diagram of the transformation in complexes **2** and **3** as a function of electron and proton addition.

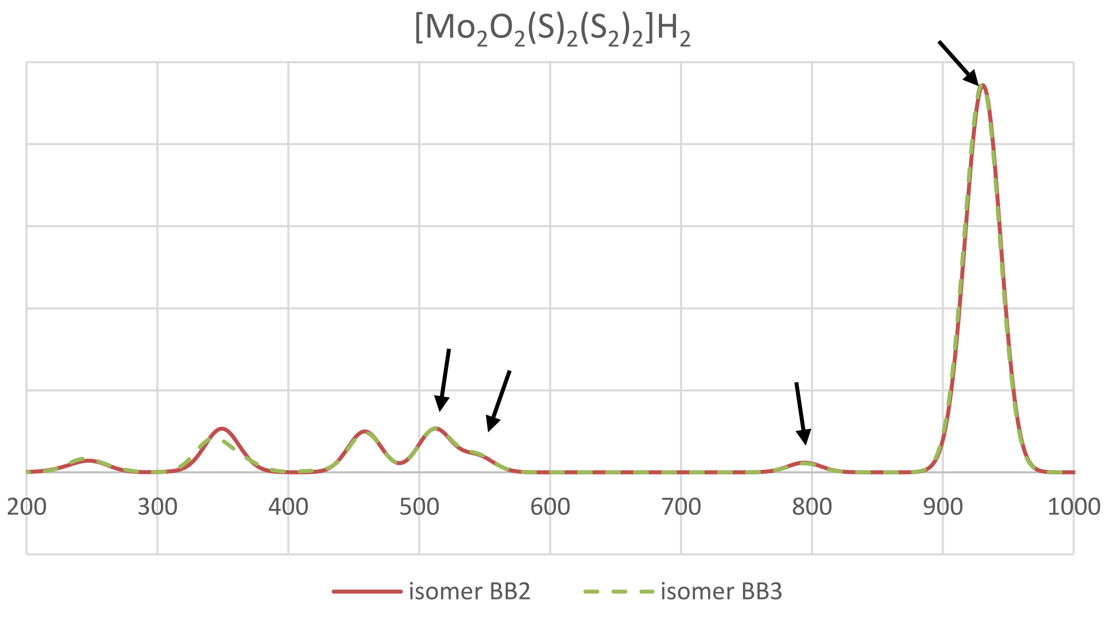

Supplementary Figure 41. Simulated Raman spectrum of (oxidised) complex **1** with relevant bands highlighted. Raman vibrational frequencies were computed at the experimental wavelength of 532nm.

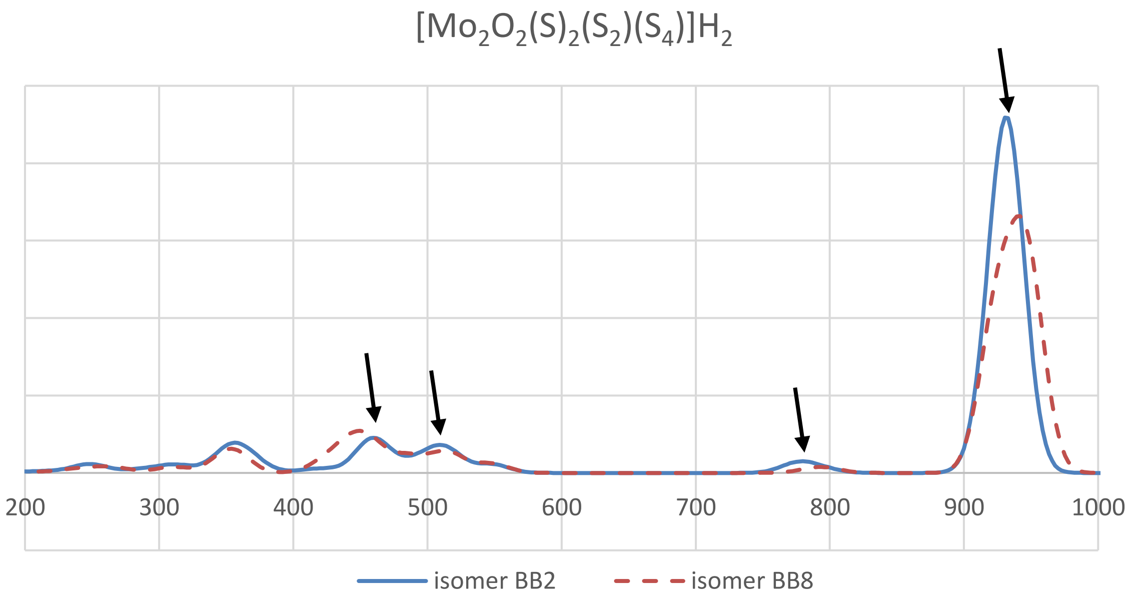

Supplementary Figure 42. Simulated Raman spectrum of (oxidised) complex **2**. Raman vibrational frequencies were computed at the experimental wavelength of 532nm.

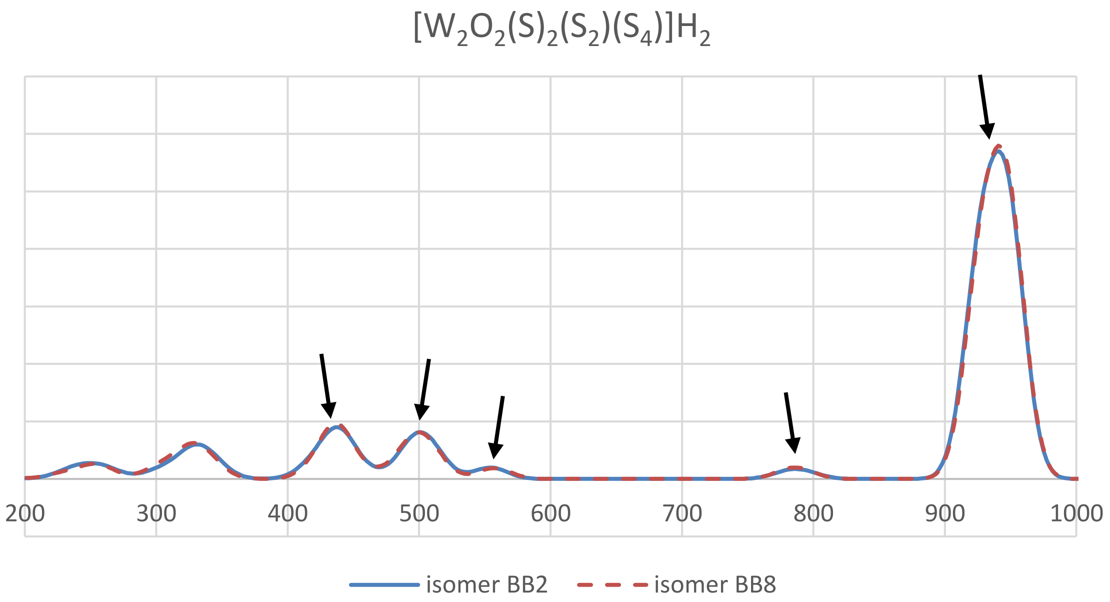

Supplementary Figure 43. Simulated Raman spectrum of (oxidised) complex **3** with relevant bands highlighted. Raman vibrational frequencies were computed at the experimental wavelength of 532nm.

Supplementary Tables

Supplementary Table 1. Catalytic ink loadings. All catalytic ink components were weighed on a six-figure balance.

$[Mo_2O_2(\mu\text{-}S)_2(S_2)_2]^{2-}$ (mg)	Carbon Powder (mg)	DMF (mL)	5% Nafion solution (µL)	Drop-cast volume (µL)	Geometric loading (µmol cm^{-2})
4.000	2.000	10	50	5	0.05
4.000	2.000	1	50	5	0.48
4.000	2.000	0.5	50	5	0.90
4.000	2.000	0.125	50	5	2.85
**$[Mo_2O_2(\mu\text{-}S)_2(S_2)(S_4)]^{2-}$ (mg)**	**Carbon Powder (mg)**	**DMF (mL)**	**5% Nafion solution (µL)**	**Drop-cast volume (µL)**	**Geometric loading (µmol cm^{-2})**
4.430	2.215	10	50	5	0.05
4.430	2.215	1	50	5	0.48
4.430	2.215	0.5	50	5	0.90
4.430	2.215	0.125	50	5	2.85
**$[W_2O_2(\mu\text{-}S)_2(S_2)(S_4)]^{2-}$ (mg)**	**Carbon Powder (mg)**	**DMF (mL)**	**5% Nafion solution (µL)**	**Drop-cast volume (µL)**	**Geometric loading (µmol cm^{-2})**
4.600	2.300	10	50	5	0.05
4.600	2.300	1	50	5	0.48
4.600	2.300	0.5	50	5	0.90
4.600	2.300	0.125	50	5	2.85

Supplementary Table 2. Crystallographic Data for $(NMe_4)_2[Mo_2O_2(\mu\text{-}S)_2(S_2)_2]$ (1), $(NMe_4)_2[Mo_2O_2(\mu\text{-}S)_2(S_2)(S_4)]$ (2), and $(NMe_4)_2[W_2O_2(\mu\text{-}S)_2(S_2)(S_4)]$ (3)

	**1**	**2**	**3**
Formula	$C_8H_{24}Mo_2N_2O_2S_6$	$C_8H_{24}Mo_2N_2O_2S_8$	$C_8H_{24}Mo_2N_2O_2S_8$
M_r [g mol^{-1}]	564.53	669.71	845.52
Temperature [K]	150(2)	150(2)	150(2)
Symmetry	Orthorhombic	Monoclinic	Monoclinic
Space group	*Pba2*	*P21/c*	*P21/c*
a [Å]	15.430(1)	20.661(7)	20.881(2)
b [Å]	24.542(1)	9.769(3)	9.778(1)
c [Å]	10.8423(8)	12.428(4)	12.338(1)
α [°]	90	90	90
β [°]	90	92.428(3)	92.612(3)
γ [°]	90	90	90
ρ_{calcd} [μg m^{-3}]	1.826	1.775	2.231
Volume [Å^3]	4106.06(50)	2506.18(150)	2516.82(50)
Z	8	4	4
μ [mm^{-1}]	1.832	1.678	9.808
F (000)	2256	1344.0	1600.0
R1[a]	0.0301	0.0442	0.0415
wR2[b]	0.0675	0.1201	0.1017
GoF, S[c]	1.071	1.057	1.279

[a] $R1 = \Sigma||F_o| - |F_c||/\Sigma|F_o|$. [b] $wR2 = \{\Sigma[w(F_o^2 - F_c^2)^2]/\Sigma[w(F_o^2)^2]\}^{1/2}$, where $w = 1/[\sigma^2(F_o^2) + (aP)^2 + bP]$, $P = (F_o^2 + 2F_c^2)/3$. [c] $GoF = \{\Sigma[w(F_o^2 - F_c^2)^2]/(n - p)\}^{1/2}$, where n = number of reflections and p is the total number of parameters refined.

Supplementary Table 3. Dissociative recombination (Tafel) step.

Catalytic species	Reactant complex	ΔG^{o}/kcal·mol^{-1}
$H_2[Mo_2O_2(\mu\text{-}S)_2(S_2)_2]^{2-} \rightarrow [Mo_2O_2(\mu\text{-}S)_2(S_2)_2]^{2-} + H_2$	BB1	+6.2
$H_2[W_2O_2(\mu\text{-}S)_2(S_2)(S_4)]^{2-} \rightarrow [W_2O_2(\mu\text{-}S)_2(S_2)(S_4)]^{2-} + H_2$	BB3	+1.1
	BB1	+4.4
$H_2[Mo_2O_2(\mu\text{-}S)_2(S_2)(S_4)]^{2-} \rightarrow [Mo_2O_2(\mu\text{-}S)_2(S_2)(S_4)]^{2-} + H_2$	BC1	+4.0
	BC2	+4.3

Theoretical Calculations and evaluation of possible intermediates

The $[Mo_2O_2(S)_2(S_2)_2]^{2-}$ e^-/H^+ addition process

Supplementary Table 4. Initial protonation steps for the $[Mo_2O_2(\mu\text{-}S)_2(S_2)_2]^{2-}$ anion.

Reactant	$[Mo_2O_2S_6]^{2-}$	$[Mo_2O_2S_6]^{4-}$
$[Mo_2O_2S_6]^{n-} + H_5O_2^+ \rightarrow A + 2H_2O$	-3.4	-12.9
$[Mo_2O_2S_6]^{n-} + H_5O_2^+ \rightarrow B + 2H_2O$	**-13.5**	**-51.1**
$[Mo_2O_2S_6]^{n-} + H_5O_2^+ \rightarrow C + 2H_2O$	-5.9	-7.9

Supplementary Table 5. Second protonation step of the oxidised and doubly reduced symmetric molybdenum species.

Reactant	$H[Mo_2O_2S_6]^{-}$	$H[Mo_2O_2S_6]^{3-}$
A + $H_5O_2^+$ → AA + $2H_2O$	+12.3	+8.0
B + $H_5O_2^+$→ BB1 + $2H_2O$	+13.1	**-41.3**
B + $H_5O_2^+$ → BB2 + $2H_2O$	**-8.9**	-16.1
B + $H_5O_2^+$ → BB3 + $2H_2O$	**-9.5**	-15.7
B + $H_5O_2^+$ →AB1 + $2H_2O$	+0.5	-27.5
B + $H_5O_2^+$ →AB2 + $2H_2O$	+2.3	-6.0
B + $H_5O_2^+$ → BC1 + $2H_2O$	+1.6	**-**
B + $H_5O_2^+$ →BC2 + $2H_2O$	+3.4	-10.7
A + $H_5O_2^+$ →AC + $2H_2O$	+12.7	+6.7
C + $H_5O_2^+$ → CC + $2H_2O$	+11.2	+25.4

The $[W_2O_2(S)_2(S_2)(S_4)]^{2-}$ e^-/H^+ addition process

Supplementary Table 6. First protonation step of the oxidised and doubly reduced tungsten species.

Reactant	$[W_2O_2S_8]^{2–}$	$[W_2O_2S_8]^{4–}$
$[W_2O_2S_8]^{n–} + H_5O_2^+ \rightarrow$ A1+ $2H_2O$	-2.2	-25.9
$[W_2O_2S_8]^{n–} + H_5O_2^+ \rightarrow$ A2+ $2H_2O$	-2.1	-28.9
$[W_2O_2S_8]^{n–} + H_5O_2^+ \rightarrow$ B1+ $2H_2O$	**-10.0**	-39.6
$[W_2O_2S_8]^{n–} + H_5O_2^+ \rightarrow$ B2+ $2H_2O$	-5.8	**-43.9**
$[W_2O_2S_8]^{n–} + H_5O_2^+ \rightarrow$ B3+ $2H_2O$	+2.8	-32.5
$[W_2O_2S_8]^{n–} + H_5O_2^+ \rightarrow$ C+ $2H_2O$	-2.4	-8.1

Supplementary Table 7. Second protonation step of the oxidised tungsten species.

Reactant	$H[W_2O_2S_8]^-$
B1 + $H_5O_2^+$ $\rightarrow$ BB1 + $2H_2O$	+14.8
B1 + $H_5O_2^+$ $\rightarrow$ BB2 + $2H_2O$	**-3.2**
B1 + $H_5O_2^+$ $\rightarrow$ BB6 + $2H_2O$	-1.5
B1 + $H_5O_2^+$ $\rightarrow$ BB7 + $2H_2O$	+4.9
B1 + $H_5O_2^+$ $\rightarrow$ BB8 + $2H_2O$	**-3.3**
B1 + $H_5O_2^+$ $\rightarrow$ AB1 + $2H_2O$	+1.4
B1 + $H_5O_2^+$ $\rightarrow$ AB2 + $2H_2O$	+0.5
B1 + $H_5O_2^+$ $\rightarrow$ BC1 + $2H_2O$	+8.9
B1 + $H_5O_2^+$ $\rightarrow$ BC2 + $2H_2O$	+6.8

Supplementary Table 8. Second protonation step of the doubly reduced tungsten species.

**Reactant**	**$H[W_2O_2S_8]^{3-}$**
B2 + $H_5O_2^+$ $\rightarrow$ BB8 + $2H_2O$	-11.7
B2 + $H_5O_2^+$ $\rightarrow$ BB2 + $2H_2O$	-12.7
B2 + $H_5O_2^+$ $\rightarrow$ BB3 + $2H_2O$	-29.2
B2 + $H_5O_2^+$ $\rightarrow$ BB4 + $2H_2O$	-16.4
B2 + $H_5O_2^+$ $\rightarrow$ BB5 + $2H_2O$	-13.9
B2 + $H_5O_2^+$ $\rightarrow$ AB3 + $2H_2O$	-2.9
B2 + $H_5O_2^+$ $\rightarrow$ AB4 + $2H_2O$	—
B2 + $H_5O_2^+$ $\rightarrow$ BC3 + $2H_2O$	-4.3
B2 + $H_5O_2^+$ $\rightarrow$ BC4 + $2H_2O$	-2.1

The $[Mo_2O_2(S)_2(S_2)(S_4)]^{2-}$ e^-/H^+ addition process

Supplementary Table 9. First protonation step of the oxidised and doubly reduced asymmetric molybdenum species.

Reactant	$[Mo_2O_2S_8]^{2-}$	$[Mo_2O_2S_8]^{4-}$
$[Mo_2O_2S_8]^{n-} + H_5O_2^+ \rightarrow A1 + 2H_2O$	-1.9	-26.7
$[Mo_2O_2S_8]^{n-} + H_5O_2+ \rightarrow A2 + 2H_2O$	-2.8	-32.8
$[Mo_2O_2S_8]^{n-} + H_5O_2^+ \rightarrow B1 + 2H_2O$	**-12.0**	**-39.7**
$[Mo_2O_2S_8]^{n-} + H_5O_2^+ \rightarrow B2 + 2H_2O$	-6.7	-36.1
$[Mo_2O_2S_8]^{n-} + H_5O_2^+ \rightarrow B3 + 2H_2O$	3.0	-35.8
$[Mo_2O_2S_8]^{n-} + H_5O_2^+ \rightarrow C + 2H_2O$	-3.7	-12.8

Supplementary Table 10. Second protonation step of the oxidised asymmetric molybdenum species.

Reactant	$H[Mo_2O_2S_8]^-$
$B1 + H_5O_2^+ \rightarrow BB1 + 2H_2O$	+14.5
$B1 + H_5O_2^+ \rightarrow BB2 + 2H_2O$	-3.3
$B1 + H_5O_2^+ \rightarrow BB6 + 2H_2O$	+0.3
$B1 + H_5O_2^+ \rightarrow BB7 + 2H_2O$	-0.8
$B1 + H_5O_2^+ \rightarrow BB8 + 2H_2O$	-3.5
$B1 + H_5O_2^+ \rightarrow AB1 + 2H_2O$	+6.2
$B1 + H_5O_2^+ \rightarrow AB2 + 2H_2O$	+6.6
$B1 + H_5O_2^+ \rightarrow BC1 + 2H_2O$	+3.9
$B1 + H_5O_2^+ \rightarrow BC2 + 2H_2O$	+1.8

Supplementary Table 11. Second protonation step of the doubly reduced asymmetric molybdenum species.

Reactant	$H[Mo_2O_2S_8]^{3-}$
B1 + $H_5O_2^+$ $\rightarrow$ BB1 + $2H_2O$	-38.8
B1 + $H_5O_2^+$ $\rightarrow$ BB2 + $2H_2O$	-21.3
B1 + $H_5O_2^+$ $\rightarrow$ BB6 + $2H_2O$	-8.7
B1 + $H_5O_2^+$ $\rightarrow$ BB7 + $2H_2O$	-9.0
B1 + $H_5O_2^+$ $\rightarrow$ BB8 + $2H_2O$	-9.9
B1 + $H_5O_2^+$ $\rightarrow$ AB1 + $2H_2O$	+15.9
B1 + $H_5O_2^+$ $\rightarrow$ AB2 + $2H_2O$	-6.4
B1 + $H_5O_2^+$ $\rightarrow$ BC1 + $2H_2O$	-38.4
B1 + $H_5O_2^+$ $\rightarrow$ BC2 + $2H_2O$	-38.6

Supplementary Table 12. Volmer steps starting from the original anions reacting with a solvated hydrogen atom.

Volmer steps	Calculated ΔG° (eV)
$[Mo_2O_2S_6]^{2-} + H\cdot \rightarrow A$	-1.21
$[Mo_2O_2S_6]^{2-} + H\cdot \rightarrow B$	**-1.66**
$[Mo_2O_2S_6]^{2-} + H\cdot \rightarrow C$	-0.88
$[W_2O_2S_8]^{2-} + H\cdot \rightarrow A1$	-0.88
$[W_2O_2S_8]^{2-} + H\cdot \rightarrow A2$	-1.12
$[W_2O_2S_8]^{2-} + H\cdot \rightarrow B1$	-1.69
$[W_2O_2S_8]^{2-} + H\cdot \rightarrow B2$	**-1.95**
$[W_2O_2S_8]^{2-} + H\cdot \rightarrow B3$	-1.54
$[W_2O_2S_8]^{2-} + H\cdot \rightarrow C$	-0.90
$[Mo_2O_2S_8]^{2-} + H\cdot \rightarrow A1$	-0.73
$[Mo_2O_2S_8]^{2-} + H\cdot \rightarrow A2$	-1.45
$[Mo_2O_2S_8]^{2-} + H\cdot \rightarrow B1$	-1.52
$[Mo_2O_2S_8]^{2-} + H\cdot \rightarrow B2$	-1.43
$[Mo_2O_2S_8]^{2-} + H\cdot \rightarrow B3$	**-1.61**
$[Mo_2O_2S_8]^{2-} + H\cdot \rightarrow C$	-0.82

Supplementary Table 13. Heyrovsky steps starting from the preceding singly reduced species.

Heyrovsky steps	Calculated ΔGº (eV)
$H[Mo_2O_2S_6]^{2-}$ (B) + H· → $[Mo_2O_2S_6]^{2-}$ + H_2	-2.92
$H[W_2O_2S_8]^{2-}$ (B2) + H· → $[W_2O_2S_8]^{2-}$ + H_2	-2.63
$H[Mo_2O_2S_8]^{2-}$ (B1) + H· → $[Mo_2O_2S_8]^{2-}$ + H_2	-2.97

Supplementary Table 14. Vibrational displacements occurring in the BB2 isomer of **1**.

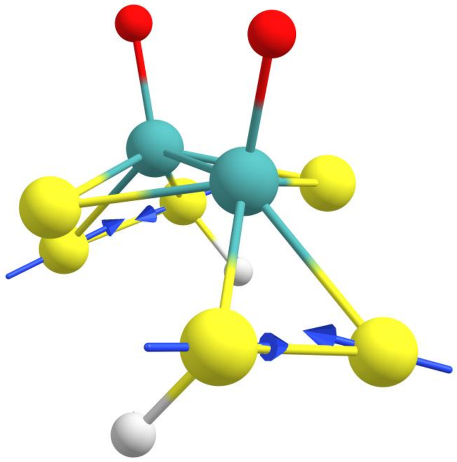 513 cm^{-1}	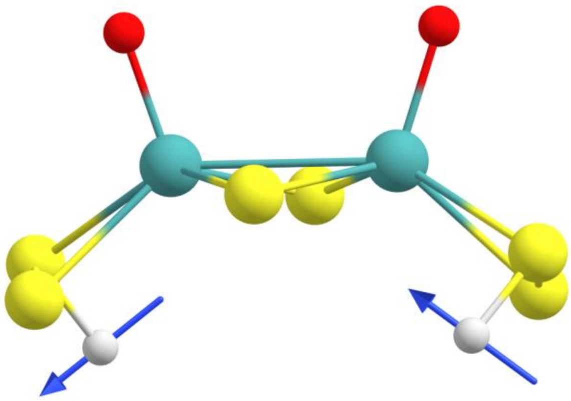 545 cm^{-1}
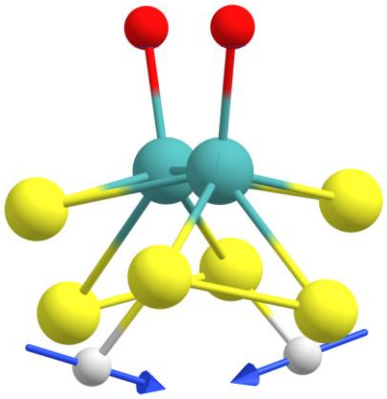 795 cm^{-1}	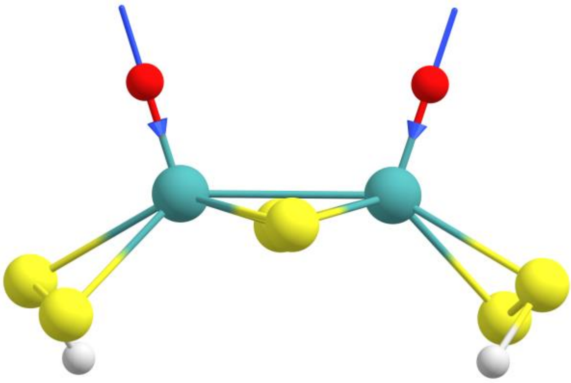 933 cm^{-1}

Supplementary Table 15. Selected vibrational modes of the BB2 isomer of **2**.

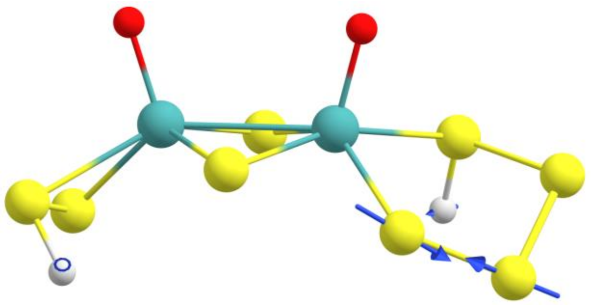<br>402 cm^{-1}	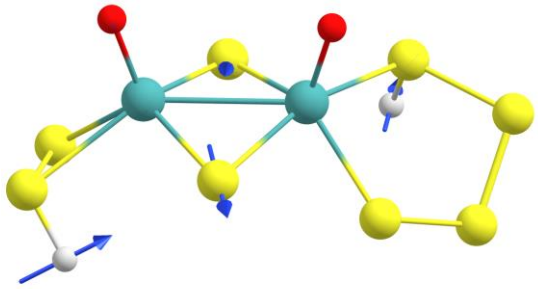<br>459 cm^{-1}
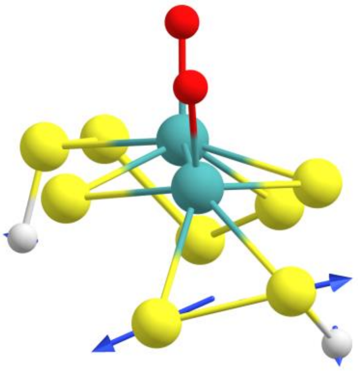<br>514 cm^{-1}	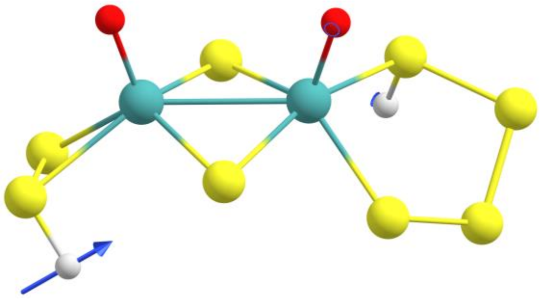<br>548 cm^{-1}
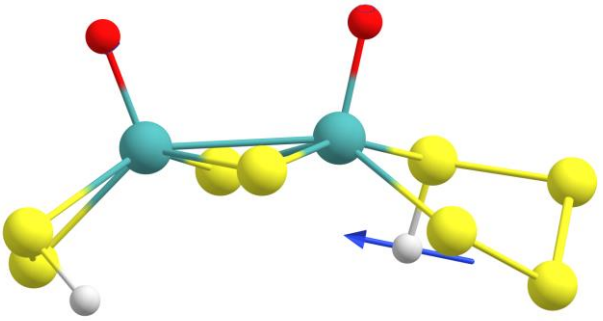<br>776 cm^{-1}	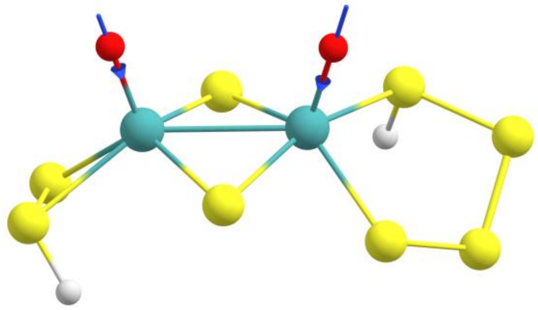<br>933 cm^{-1}

Supplementary Table 16. Selected vibrational modes of the BB2 isomer of **3**.

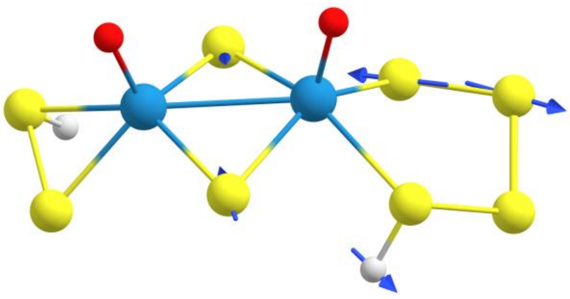 421 cm^{-1}	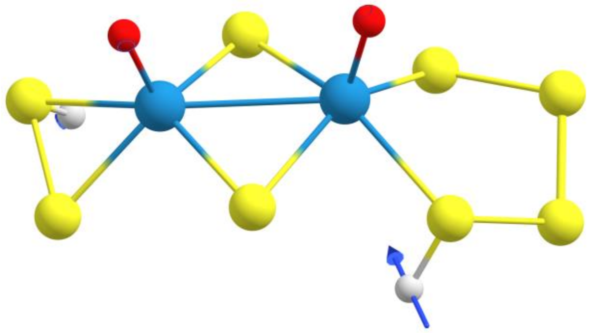 462 cm^{-1}
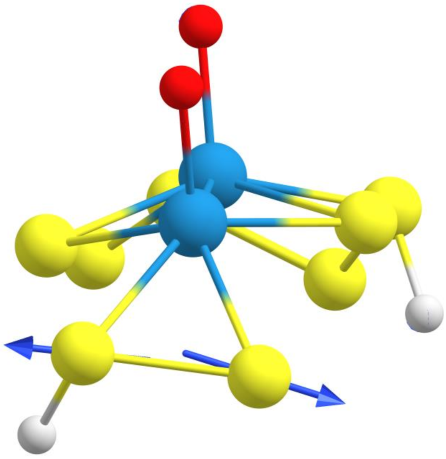 509 cm^{-1}	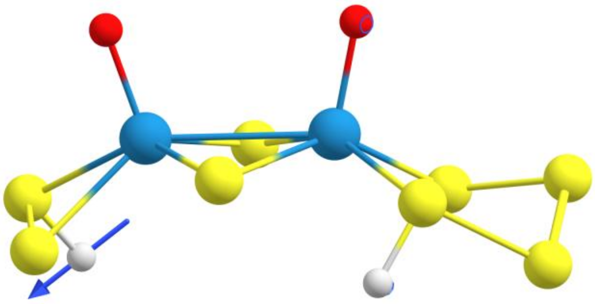 554 cm^{-1}
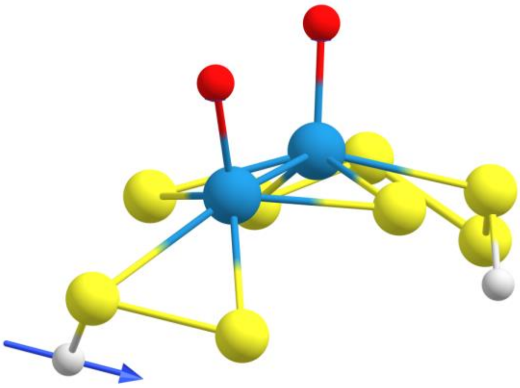 794 cm^{-1}	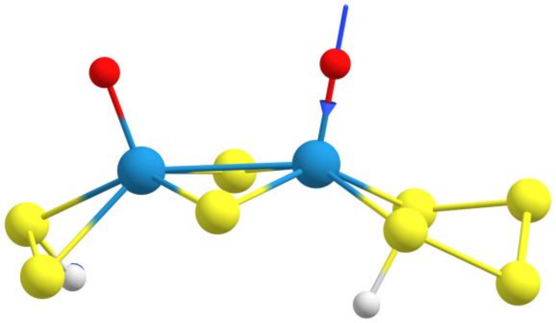 948cm^{-1}

Supplementary Notes

Supplementary Note 1

The relative changes of the intensity or broadening and overlapping of the peaks observed in Supplementary Figures 14 - 16 is quite normal specifically in Raman spectroscopy due to solvent effects which affect the relevant stretching and bending frequencies to different extend. Specifically, trapped water molecules under the nafion film used for the preparation of the catalytic ink, develop hydrogen bonds which affect the intensity of the peaks or the minor modification of spectral features as reported previously.[1,2]

Moreover, in an effort to determine the reason of absence of two peaks in the Raman spectrum of compound **3** (Supplementary Figure 16) we calculated theoretically the Raman spectra of all the three complexes and identified exactly which frequency correspond to which bond vibration (see Supplementary Figures 41 - 43 and Supplementary Tables 14 - 16). For example, the missing peak in the Raman region of 500 cm^{-1} and appearance of broad low intensity in the region of 850 cm^{-1} is due to the hydrogen bonds developed between the S terminal groups and weakening of the S···S bond (missing peak).

Additionally, after the catalytic procedure we extracted the molecular species in organic solvent and investigated again the speciation in solution using high resolution electrospray ionization mass spectrometry (see Supplementary Figures 17-19). From the mass spectrometry we were also able to identify a number of protonated species which have been predicted by the theory.

Supplementary Note 2

Cyclic voltammograms of the surface attached catalyst were recorded in organic medium and compared with the CVs of freshly prepared complexes in solution (Supplementary Figures 20-22). More specifically, a degassed solution of tetrabutylammonium tetrafluoroborate (TBA-BF_4) in dry acetonitrile (25 mL, 0.2 mol L^{-1}) was used as the electrolyte solution. The cyclic voltammograms of **1**, **2**, and **3** were recorded by dissolving 25 mg of fresh catalyst in the electrolyte solution. Cyclic voltammetry measurements were then recorded between 0 and – 1 V (vs Ag │ Ag^+) at a scan rate of 50 mV sec^{-1}. To determine the stability of the catalyst, fresh catalytic inks of **1**, **2**, and **3** were drop-cast onto glassy carbon electrodes and cycled 1000 times in acidic conditions (as-described previously). The cycled electrodes were left to dry at room temperature. The redox potentials of the used catalysts were then measured using cyclic voltammetry in TBA-BF_4/acetonitrile electrolyte solution under the same conditions described above. The figures below compare the recorded CVs for catalysts' **1**, **2**, and **3** before and after 1000 cycles. As expected, in the absence of H^+ source, no catalytic effect is observed within the potential window. Additionally, the CVs shows identical features which demonstrates the stability of the catalyst under the reported experimental conditions.

Supplementary Note 3

Investigation of Tafel-type re-combination step

The initial set of calculations were crucial in our effort to identify the most energetically favourable chemical pathway *via* the proton addition can take place (Supplementary Figure 25). The second stage of our theoretical work was focused on the thermodynamics of the electrochemical steps involved by comparing the obtained free energies of the reduced species with the relevant ones obtained for the parent compounds. All these values are summarized in relevant tables in the Supplementary Tables 3-13. The proposed protonation pathways in all the catalysts, points towards a Tafel-type recombination step due to the proximity of the neighbouring hydrogen atoms in the doubly protonated intermediates. However, it is observed that this step is endergonic for all the systems as shown in Supplementary Table 3.

Despite the endergonicity, this step it may still be viable kinetically, and as the main product is in the gas phase and is removed from the chemical system, its equilibrium may be displaced towards the desirable direction according to Le Chatelier's principle. In order to investigate further this assumption, we calculated the transition states pertaining to the Tafel steps for all three systems (Supplementary Table 3).

The transition states for the two molybdenum systems $H_2[Mo_2O_2(\mu\text{-}S)_2(S_2)_2]^{2-}$ and $H_2[M_2O_2(\mu\text{-}S)_2(S_2)(S_4)]^{-}$ (M=Mo,W) have prohibitively high activation energies to be operative at room temperature (+73.2 and +70.6 kcal·mol^{-1} respectively). The tungsten system has a lower activation energy (+42.1 kcal·mol^{-1}) but still well below the +25 kcal·mol^{-1} threshold of room temperature activation. The reason for the obtained high values is enthalpic since the entropies of activation are very small ($\Delta S^{\ddagger}$ = –2.0 cal·mol^{-1}·K^{-1} for $H_2[Mo_2O_2(\mu\text{-}S)_2(S_2)_2]^{2-}$ and $\Delta S^{\ddagger}$ = +5.2 cal·mol^{-1}·K^{-1} for $H_2[W_2O_2(\mu\text{-}S)_2(S_2)(S_4)]^{2-}$).

More specifically, in the case of BB1 intermediate, a new S-S bond needs to be reformed in tandem with the S-H bond breaking (Fig. 7). This is no longer the case with the tungsten based BB3 intermediate species, since the sulfur atoms are located far apart (3.79 Å) in this transition state and there is no evidence of overlapping population between them. However, any marginal displacement towards the direction of the products, will lead to a spontaneous reforming of the

S-S bond in structure BB3 since no unbound intermediate was obtained following hydrogen release. The S-H bonds however are noticeably stronger in BB1 versus BB3 which accounts for the higher activation barriers in the former. The Mulliken overlap populations are a reasonable benchmark of the S-H bond's strength, where in the case of $H_2[Mo_2O_2(\mu\text{-}S)_2(S_2)_2]^{2-}$ found to be 0.352 and in $H_2[W_2O_2(\mu\text{-}S)_2(S_2)(S_4)]^{2-}$ the relevant value was 0.328 for the metal bound sulfur and 0.341 for its counterpart, respectively.

In a similar manner, the two e^- reduction of the tungsten based oxoanion induces the dissociation of the S-S bond, but rather than having the terminal S^{2-} ligand reduced the electron pair will instead reside on the $\kappa^{1,4}$-S_4^{2-} chelate. Due to the asymmetry of the non-bridging ligands the energetics of bond breaking in the doubly reduced non-hydrogenated anions was screened and the obtained results are listed above in Supplementary Figure 25. The most stable intermediate structure in the first generation of proton additions was found to be the B2 species through which one anionic sulfur is capped thus helping to alleviate the charge generated by the newly formed anionic sites. The lowest energy pathway for additional protonation likewise was pursued through B2 which leads to isomer BB3 as the most stable species by a significant margin (12.8 $kcal \cdot mol^{-1}$) since it involves the capping of the two negative sulphur atoms that are formed during the reduction process. This again hints at a possible Tafel-type mechanism at play.

On the other hand, the molybdenum-based analogue of the asymmetric oxoanion promotes the formation of similar structures upon reduction but with slightly different energetics as shown in Supplementary Figure 25. Interestingly, the more stable isomeric derivative formed upon reduction of compound **2**, is the B1 species which is the analogue of compound **1**'s reduction as the same persulfide bond is cleaved. In a similar manner, the two e^- will preferentially dissociate the S-S bond of the terminal S^{2-} ligand. The second protonation step leads towards the formation of the degenerate set of BB1, BC1 and BC2 species which are energetically very close to each other.

Supplementary Note 4

On the first-tier, structures **A** and **C** represented in Supplementary Figure 28, might be considered to be formal Mo^{IV} species as the sulfur bonds remain. However, their electronic structure is different. In **A** there is a clear electron pair localisation on the $4d_{\pi}$ orbital in the molybdenum centre where the oxo ligand is protonated, which signifies a Mo^{V}/Mo^{III} type of species.

With **C** a Mo=Mo bond is present corresponding to the occupation of the LUMO+1 in Figure 6 of the main text and is thus a genuine Mo^{IV}/Mo^{IV} oxoanion. **C** is a mixed valence species of class III according to the Robin-Day classification whereas **A** is class II in relation to the additional two electrons added to the system. This is borne out by the gross Mulliken populations of the relevant metallic 4*d* contribution in the newly occupied MOs (53% (4*d*) of Mo^{III} + 9% (4*d*) of Mo^{V} in **A**; 22% (4*d*) +22% (4*d*) of both Mo^{IV} sites in **C**). All the structures were successfully optimised but **BC1** which in this case always led to the formation of the **BB1** isomer.

Supplementary References

1. Ethève, J., Huguet, P., Innocent, C., Bribes, J. L. & Pourcelly, G. Electrochemical and Raman Spectroscopy Study of a Nafion Perfluorosulfonic Membrane in Organic Solvent−Water Mixtures. *J. Phys. Chem. B*, **105**, 4151-4154 (2001).

2. Kunimatsu, K., Bae, B., Miyatake, K., Uchida, H., & Watanabe, M. ATR-FTIR Study of Water in Nafion Membrane Combined with Proton Conductivity Measurements during Hydration/Dehydration Cycle. *J. Phys. Chem. B*, **115**, 4315-4321 (2011).
