# Supplementary material for: Tuning and mechanistic insights of metal chalcogenide molecular catalysts for the hydrogen-evolution reaction
Source: Nat Commun. 2019 Jan 22;10:370. doi: 10.1038/s41467-018-08208-4 (PMC6342911; doi:10.1038/s41467-018-08208-4)
Supplement: Supplementary file 2 — Description of Additional Supplementary Files [file 41467_2018_8208_MOESM2_ESM.pdf]

Description of Additional Supplementary Files

File Name: Supplementary Data 1

Description: Raw txt files containing LSV data for compound **1**: Series of runs at various loadings as exported from the potentiostat and before converting current to current density. Potential here is recorded vs Ag/AgCl. In the main text the potential is reported vs NHE.

File Name: Supplementary Data 2

Description: Raw txt files containing LSV data for compound **2**: Series of runs at various loadings as exported from the potentiostat and before converting current to current density. Potential here is recorded vs Ag/AgCl. In the main text the potential is reported vs NHE.

File Name: Supplementary Data 3

Description: Raw txt files containing LSV data for compound **3**: Series of runs at various loadings as exported from the potentiostat and before converting current to current density. Potential here is recorded vs Ag/AgCl. In the main text the potential is reported vs NHE.
